# Supplementary material for: A novel biosensor for the spatiotemporal analysis of STING activation during innate immune responses to dsDNA
Source: EMBO J. 2025 Feb 21;44(7):2157–82. doi: 10.1038/s44318-025-00370-y (PMC11962129; doi:10.1038/s44318-025-00370-y)
Supplement: Supplementary file 6 — Movie EV5 [file 44318_2025_370_MOESM6_ESM.zip › Movie EV5.docx]

Movie EV5: HEK293T biosensor cells co-cultured at time = 0 with cGAS-mCherry transfected HEK293T cells (1h/frame)
