# Supplementary material for: A novel biosensor for the spatiotemporal analysis of STING activation during innate immune responses to dsDNA
Source: EMBO J. 2025 Feb 21;44(7):2157–82. doi: 10.1038/s44318-025-00370-y (PMC11962129; doi:10.1038/s44318-025-00370-y)
Supplement: Supplementary file 11 — Movie EV10 [file 44318_2025_370_MOESM11_ESM.zip › Movie EV10.docx]

Movie EV10: HeLa SIRF biosensor H2B-mCherry cells treated at time = 0 with 2 μM diABZI (1h/frame). Green: biosensor; Magenta: mCherry; Note that the DIC channel shows no impact on cell viability during treatment and recording.
