# Supplementary figures and images for: A novel biosensor for the spatiotemporal analysis of STING activation during innate immune responses to dsDNA
Source: EMBO J. 2025 Feb 21;44(7):2157–82. doi: 10.1038/s44318-025-00370-y (PMC11962129; doi:10.1038/s44318-025-00370-y)

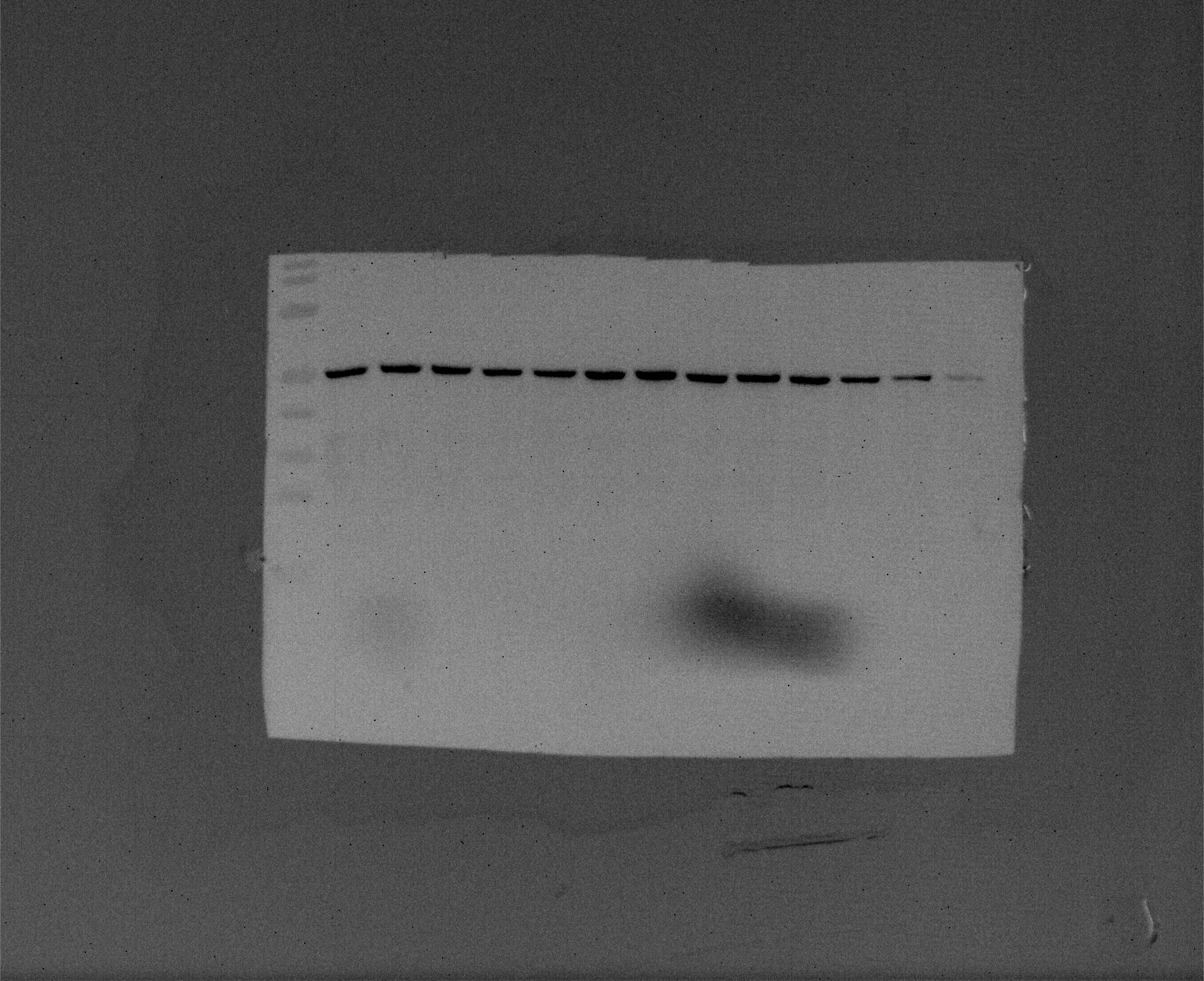

Supplement: Supplementary file 12 — Source data Fig. 1 [file 44318_2025_370_MOESM12_ESM.zip › Figure 1/Fig 1C/alpha tubulin.jpg]

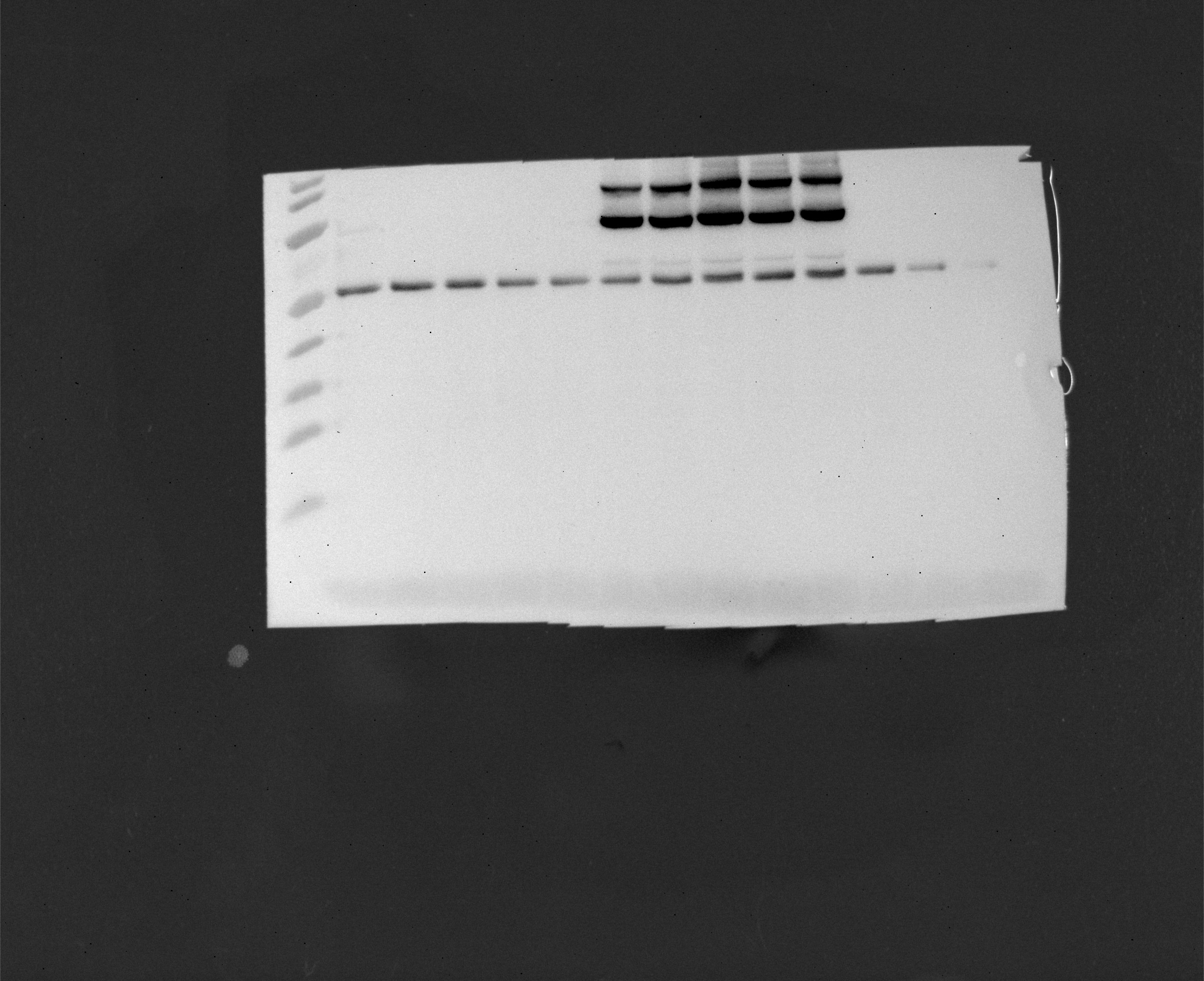

Supplement: Supplementary file 12 — Source data Fig. 1 [file 44318_2025_370_MOESM12_ESM.zip › Figure 1/Fig 1C/IRF3.jpg]

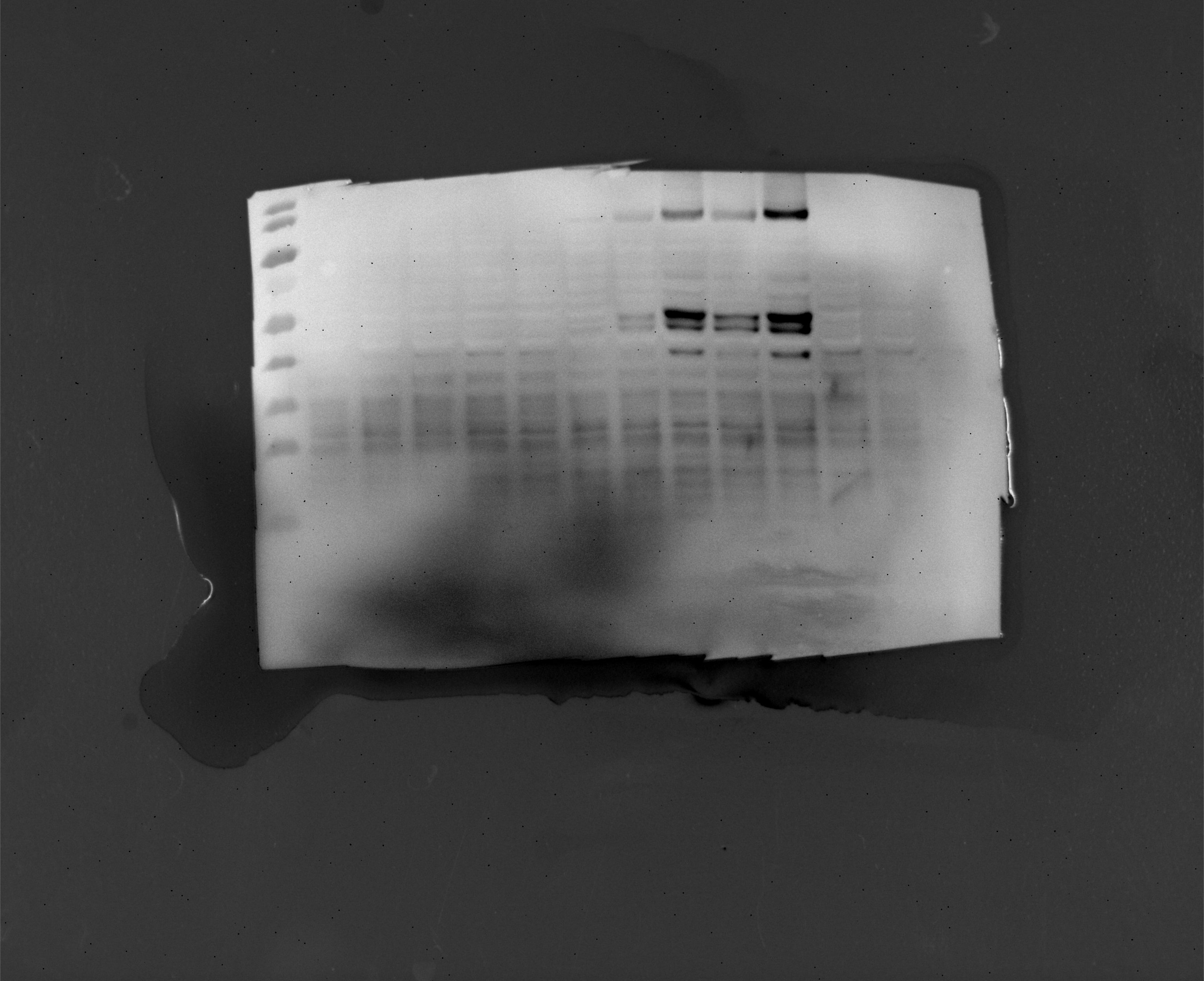

Supplement: Supplementary file 12 — Source data Fig. 1 [file 44318_2025_370_MOESM12_ESM.zip › Figure 1/Fig 1C/pSTING.jpg]

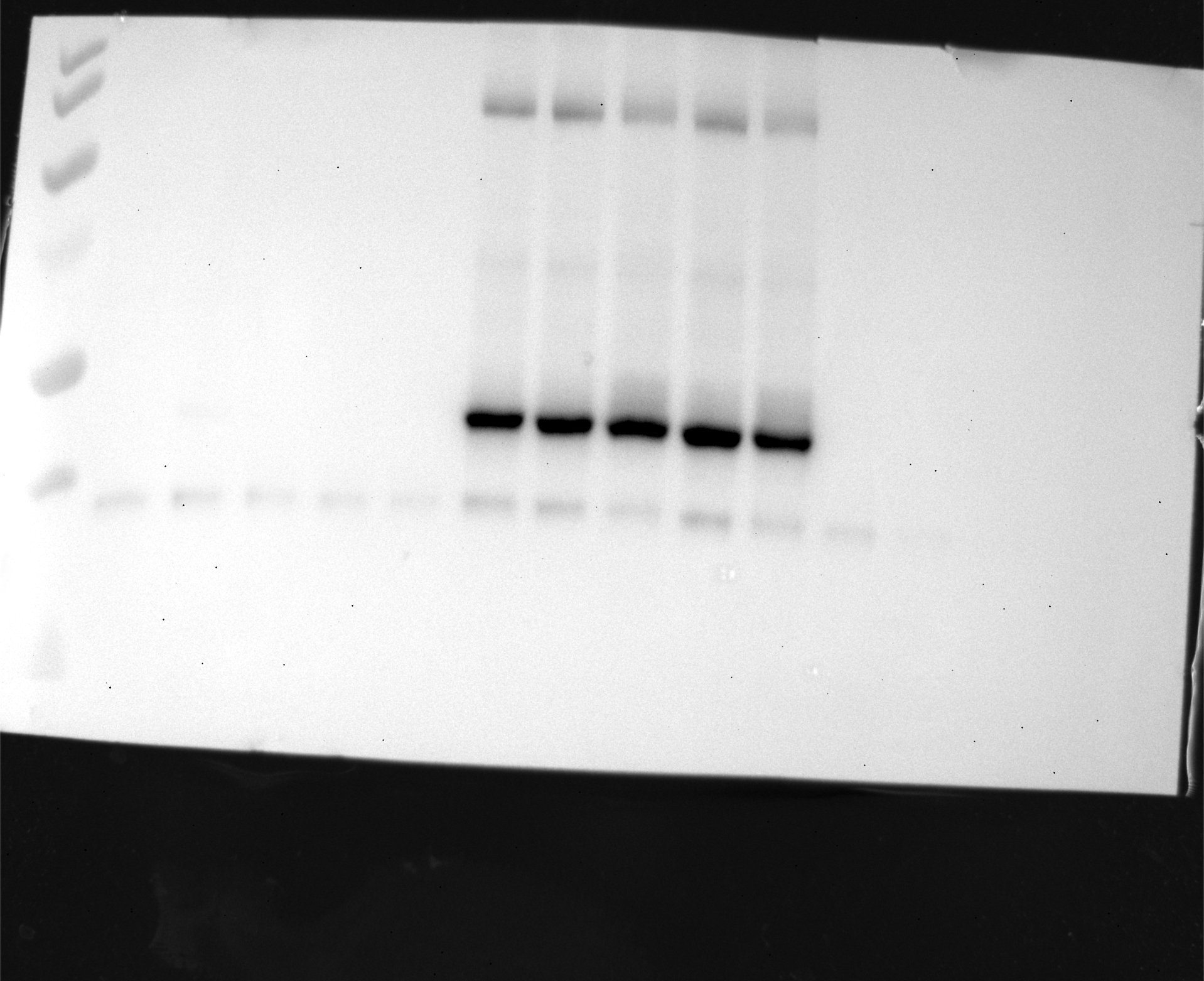

Supplement: Supplementary file 12 — Source data Fig. 1 [file 44318_2025_370_MOESM12_ESM.zip › Figure 1/Fig 1C/STING.jpg]

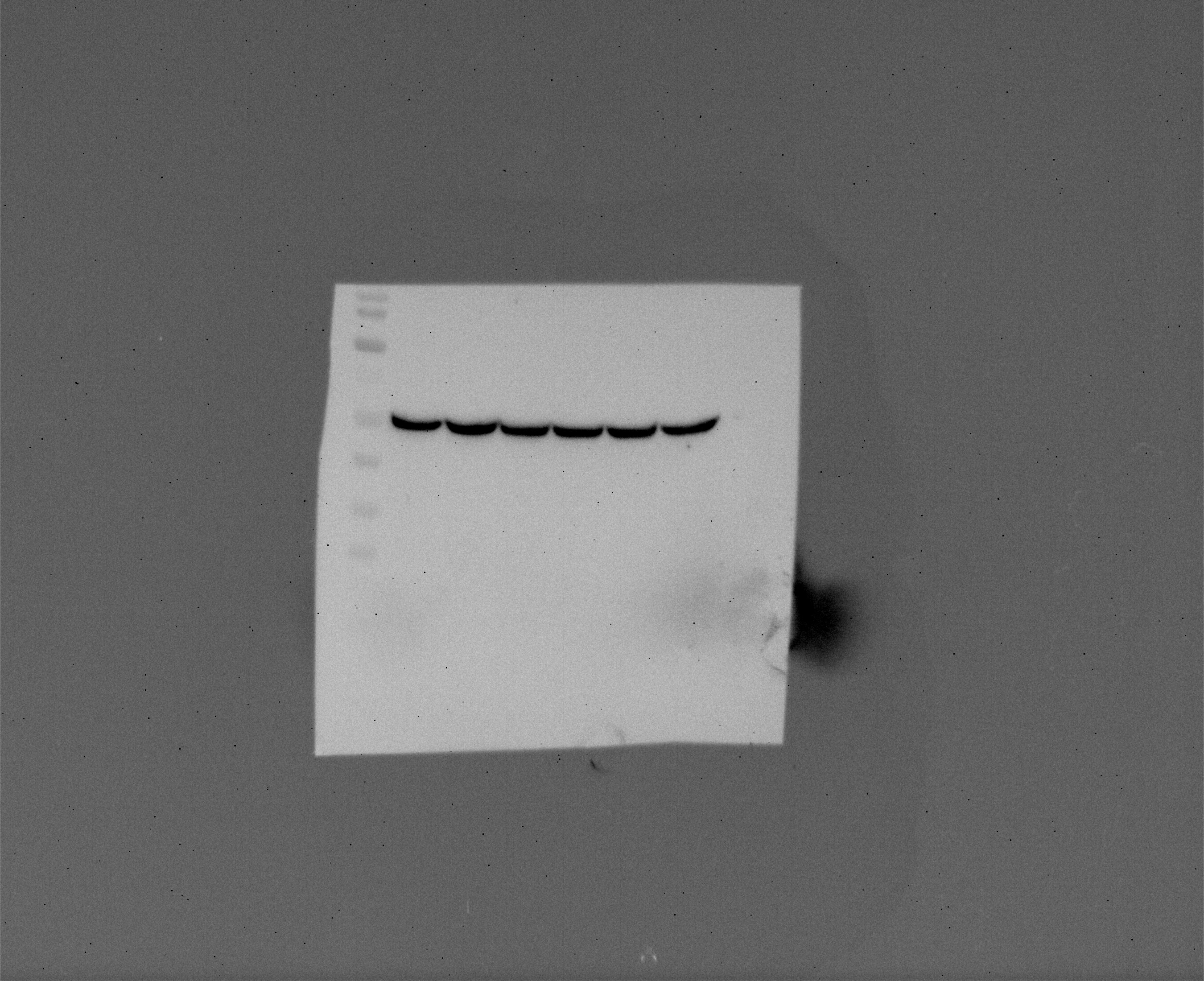

Supplement: Supplementary file 13 — Source data Fig. 2 [file 44318_2025_370_MOESM13_ESM.zip › Figure 2/Fig 2C/a-tub.jpg]

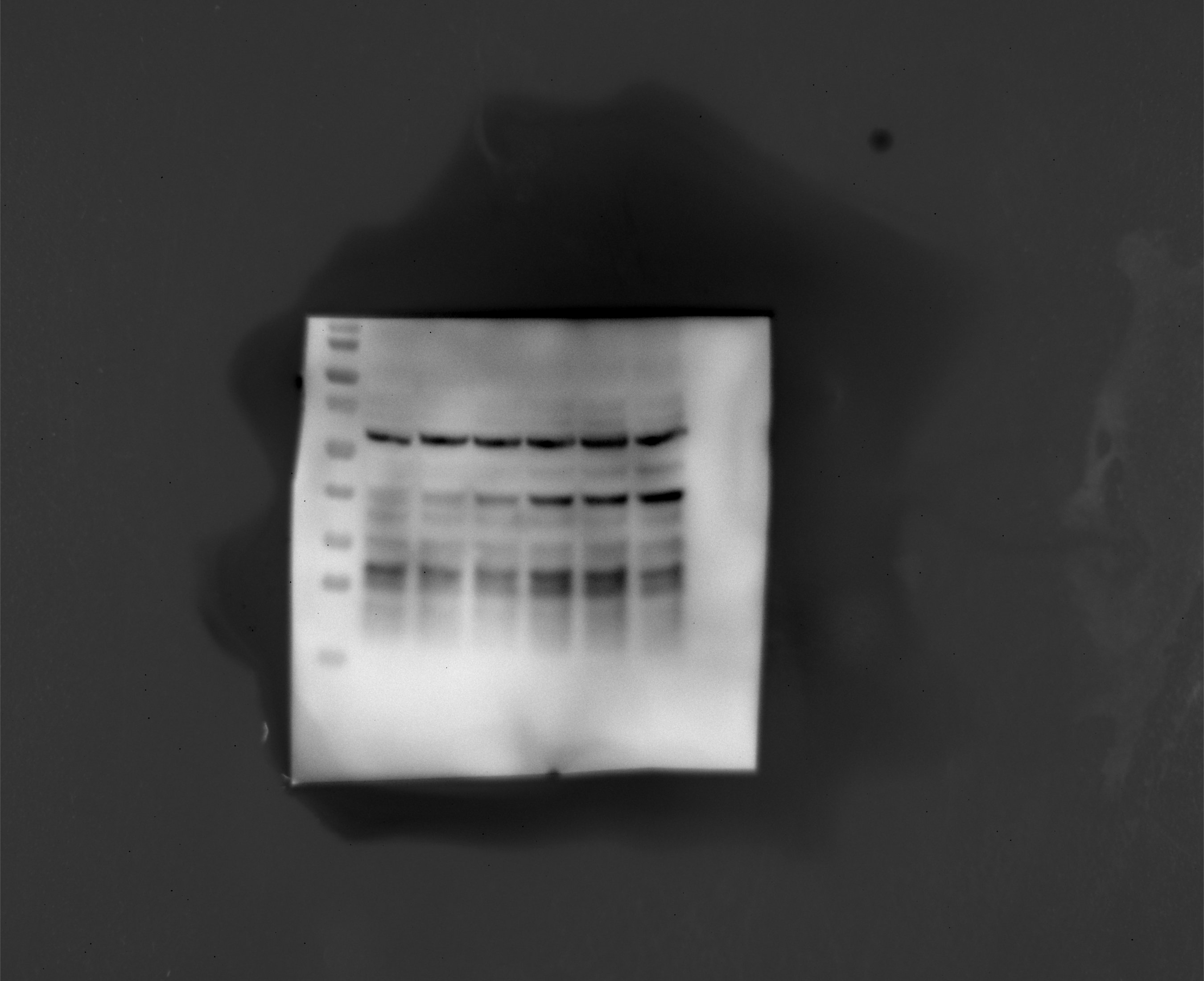

Supplement: Supplementary file 13 — Source data Fig. 2 [file 44318_2025_370_MOESM13_ESM.zip › Figure 2/Fig 2C/pSTING.jpg]

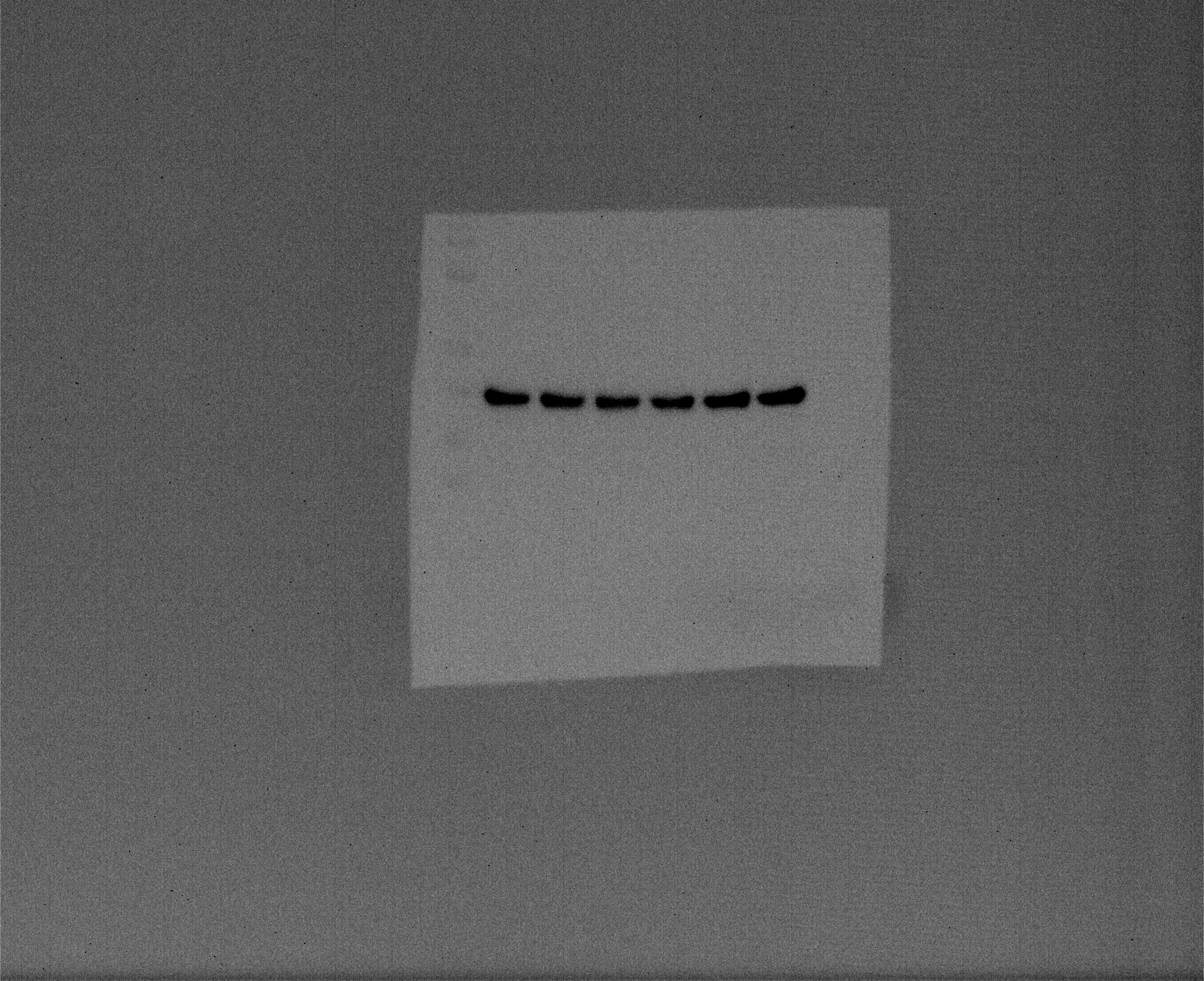

Supplement: Supplementary file 13 — Source data Fig. 2 [file 44318_2025_370_MOESM13_ESM.zip › Figure 2/Fig 2C/STING.jpg]

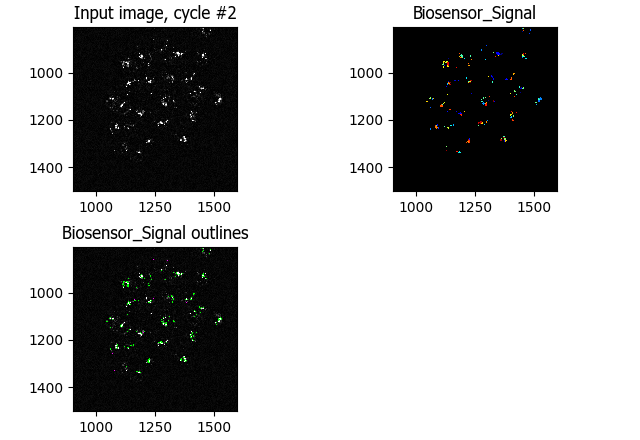

Supplement: Supplementary file 13 — Source data Fig. 2 [file 44318_2025_370_MOESM13_ESM.zip › Figure 2/Fig 2D/Biosensor.png]

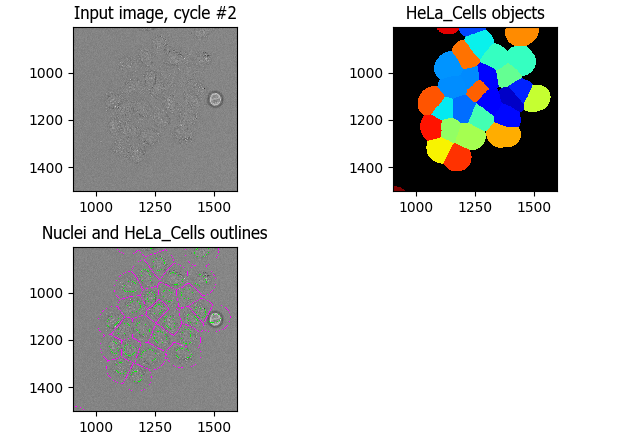

Supplement: Supplementary file 13 — Source data Fig. 2 [file 44318_2025_370_MOESM13_ESM.zip › Figure 2/Fig 2D/HeLa_Cells.png]

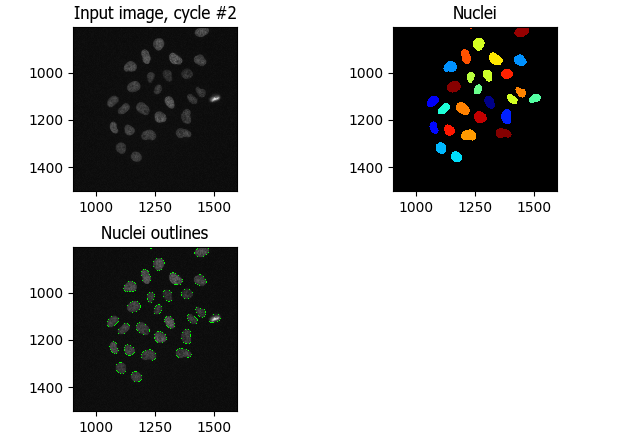

Supplement: Supplementary file 13 — Source data Fig. 2 [file 44318_2025_370_MOESM13_ESM.zip › Figure 2/Fig 2D/Nuclei.png]

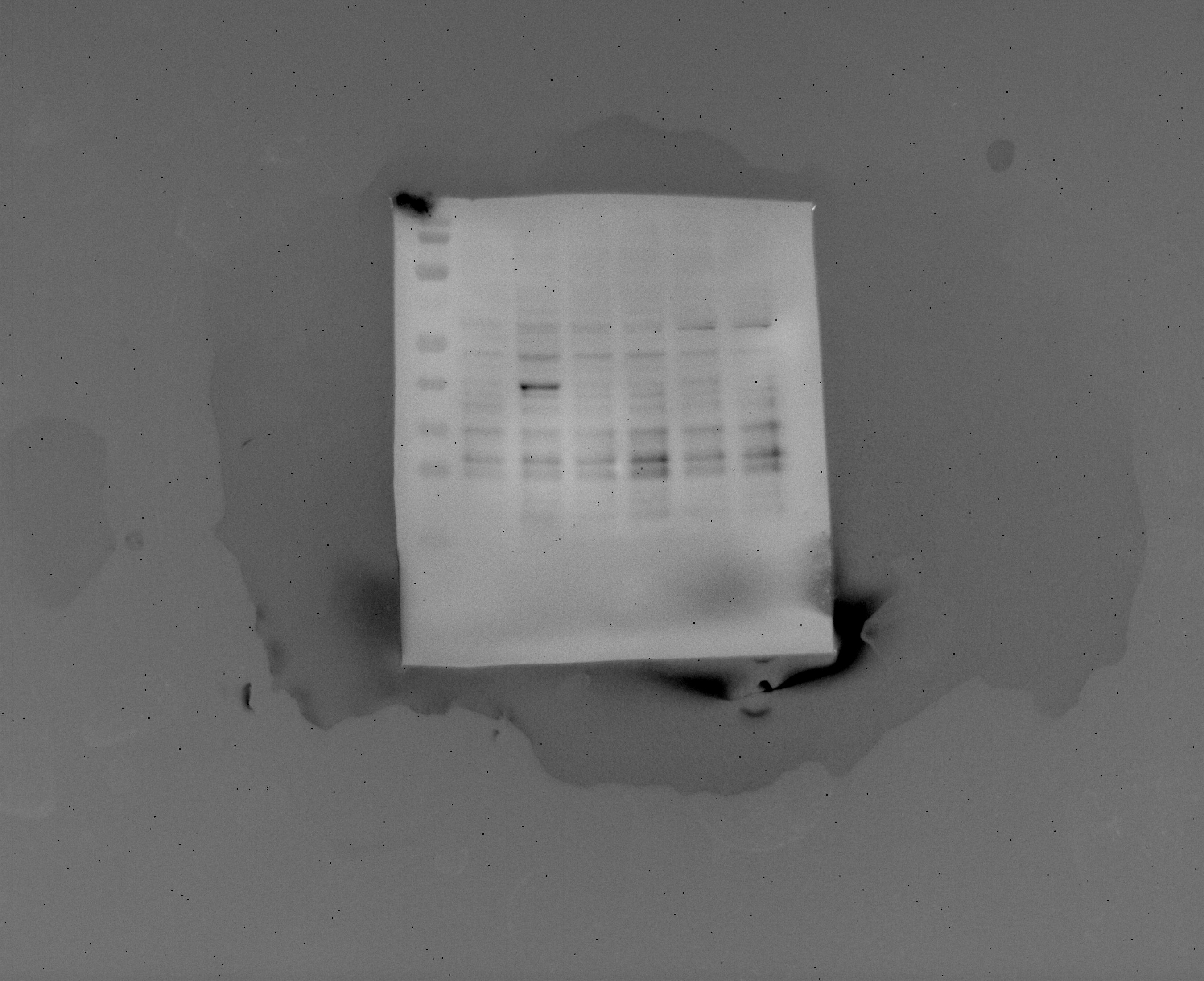

Supplement: Supplementary file 17 — Source data Fig. 6 [file 44318_2025_370_MOESM17_ESM.zip › Figure 6/Fig 6G/pSTING.jpg]

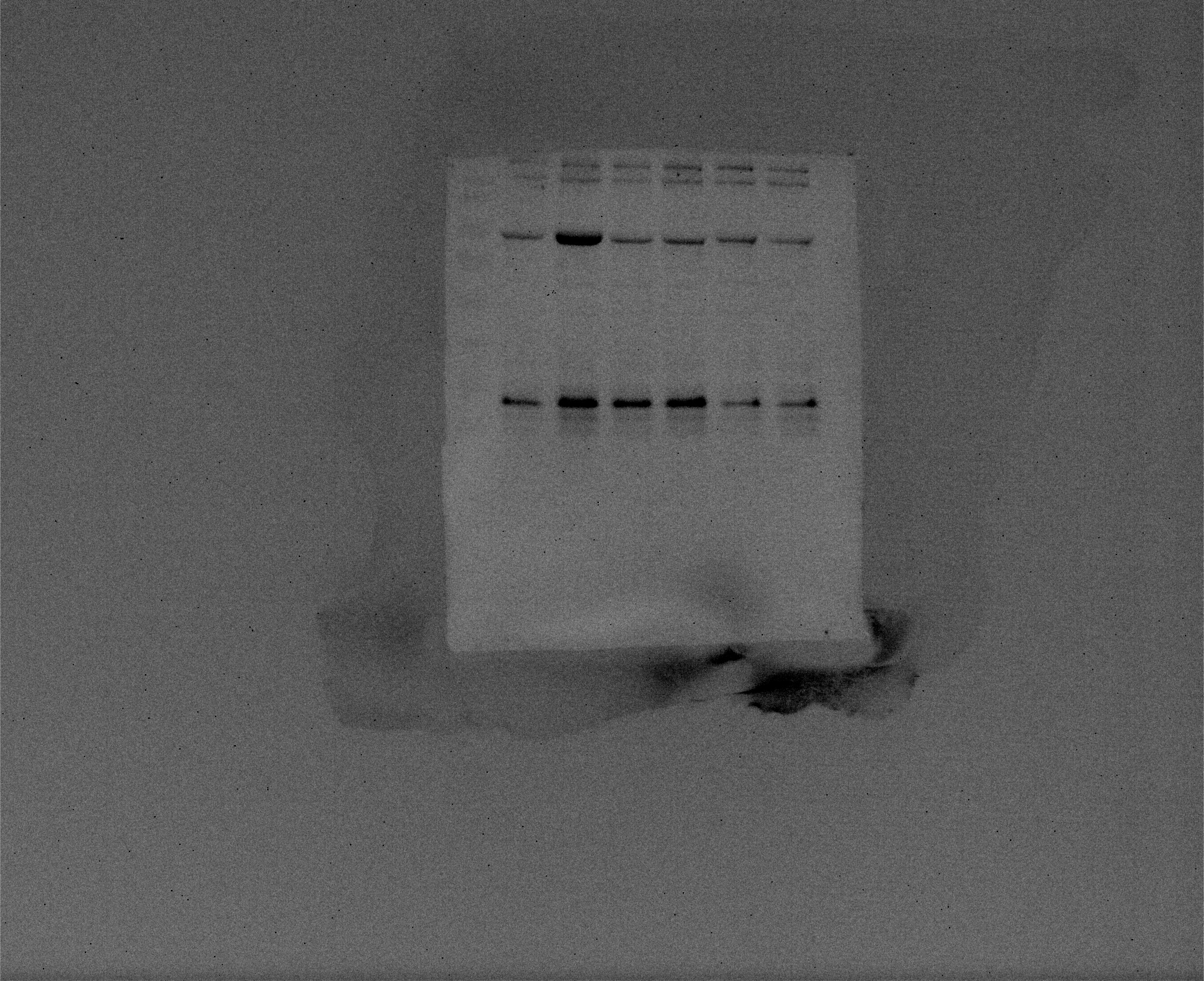

Supplement: Supplementary file 17 — Source data Fig. 6 [file 44318_2025_370_MOESM17_ESM.zip › Figure 6/Fig 6G/pTBK1.jpg]

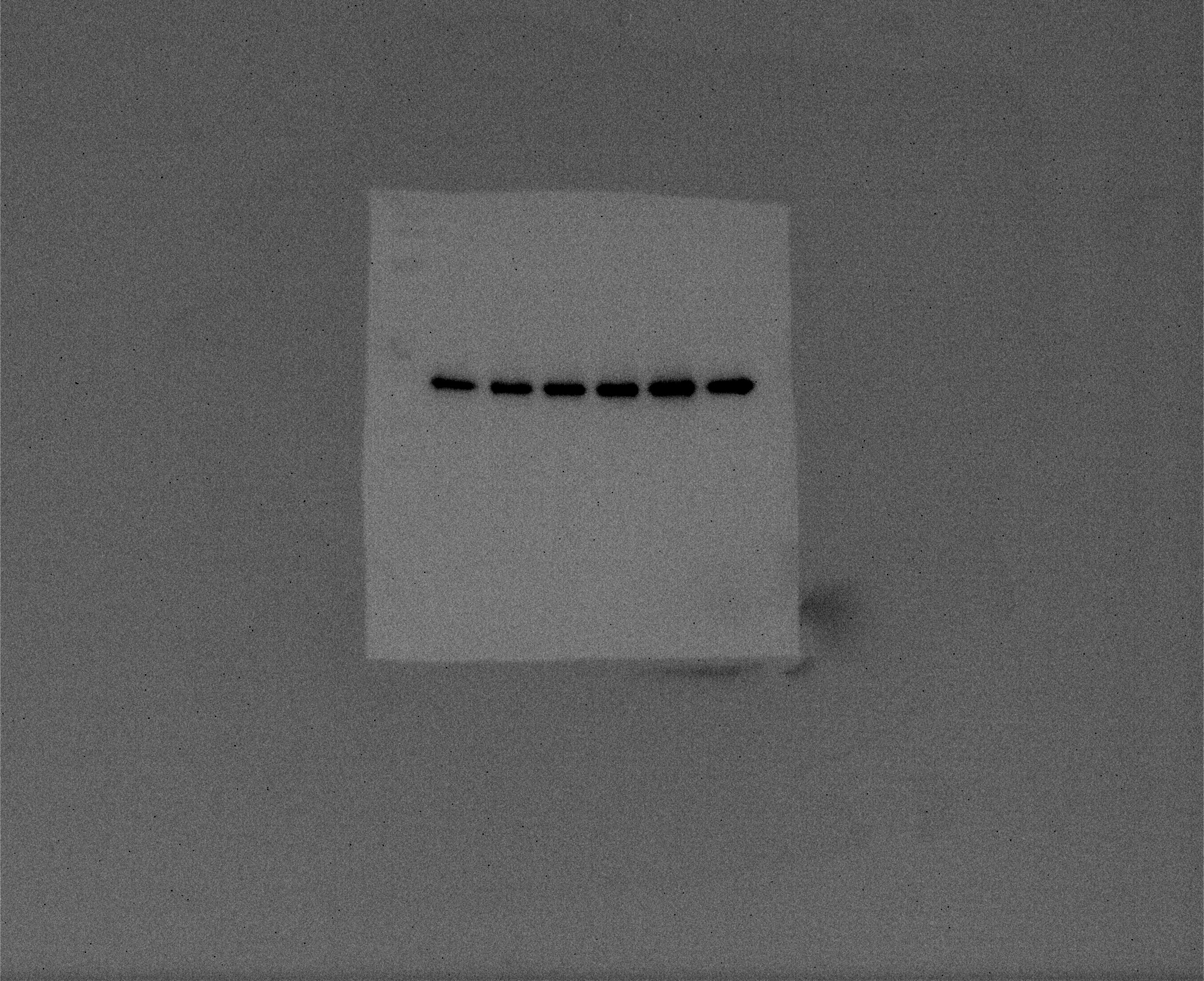

Supplement: Supplementary file 17 — Source data Fig. 6 [file 44318_2025_370_MOESM17_ESM.zip › Figure 6/Fig 6G/STING.jpg]

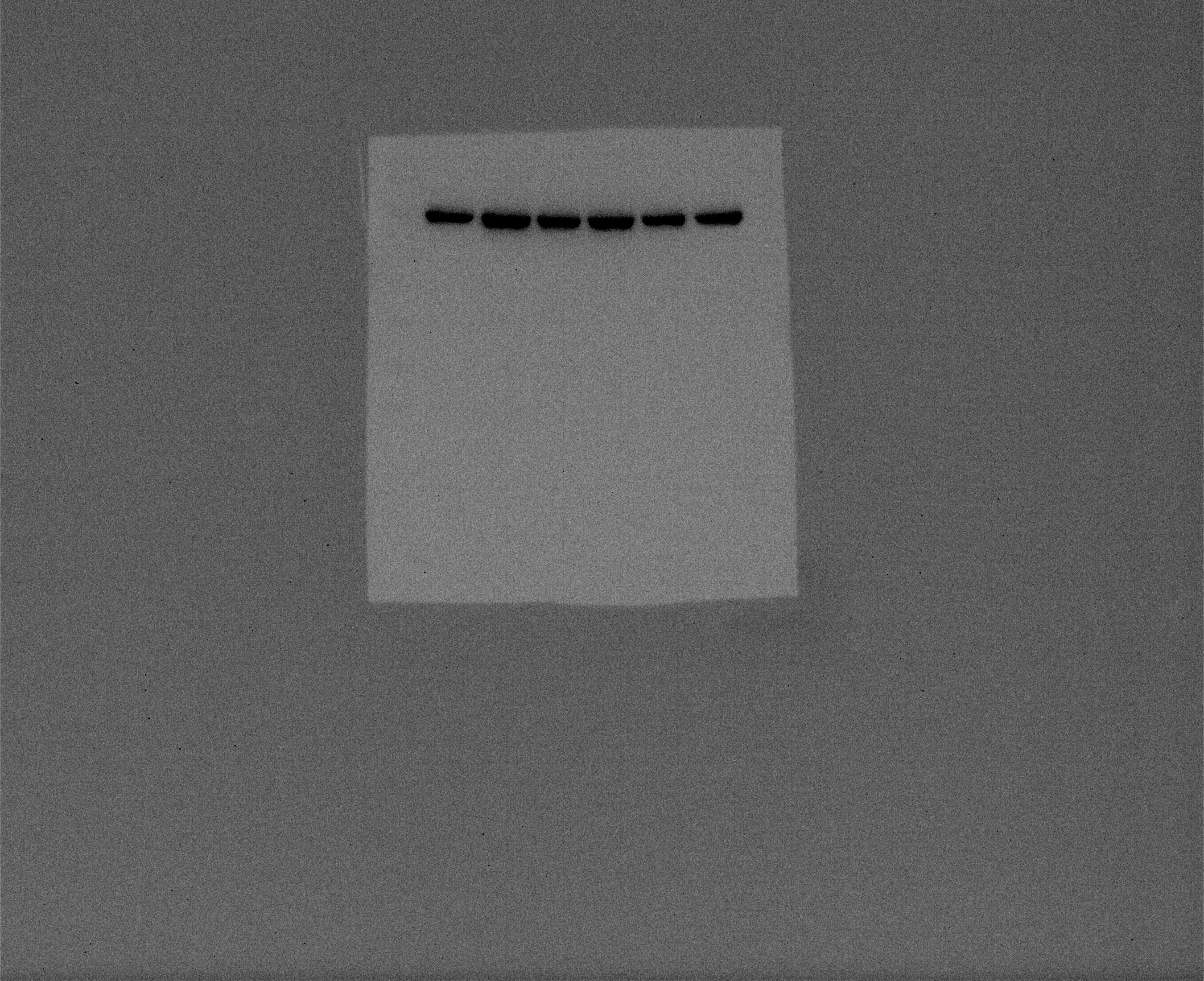

Supplement: Supplementary file 17 — Source data Fig. 6 [file 44318_2025_370_MOESM17_ESM.zip › Figure 6/Fig 6G/TBK1.jpg]

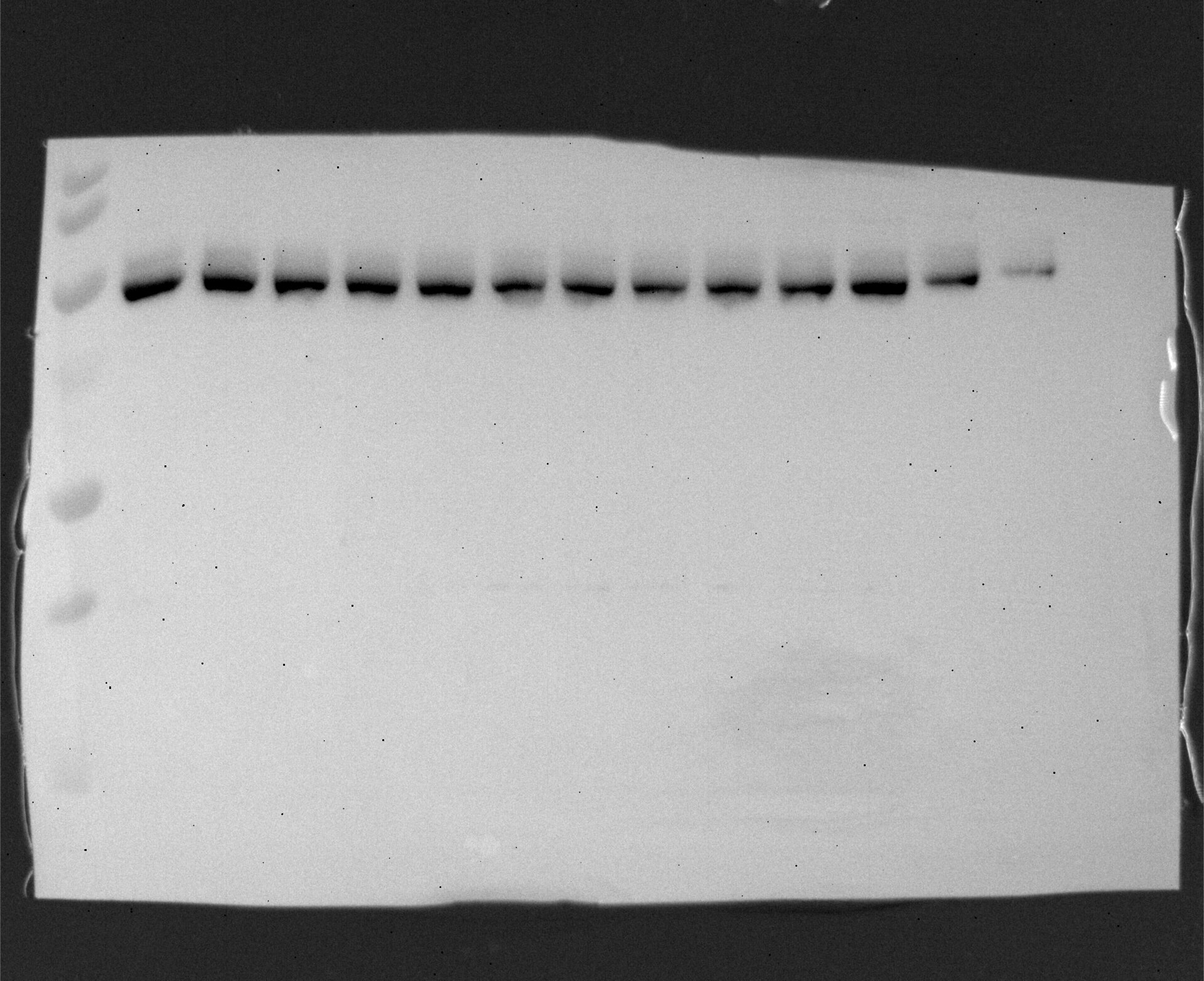

Supplement: Supplementary file 18 — EV Figure Source Data [file 44318_2025_370_MOESM18_ESM.zip › Figure EV1/Fig EV1B/TBK1.jpg]

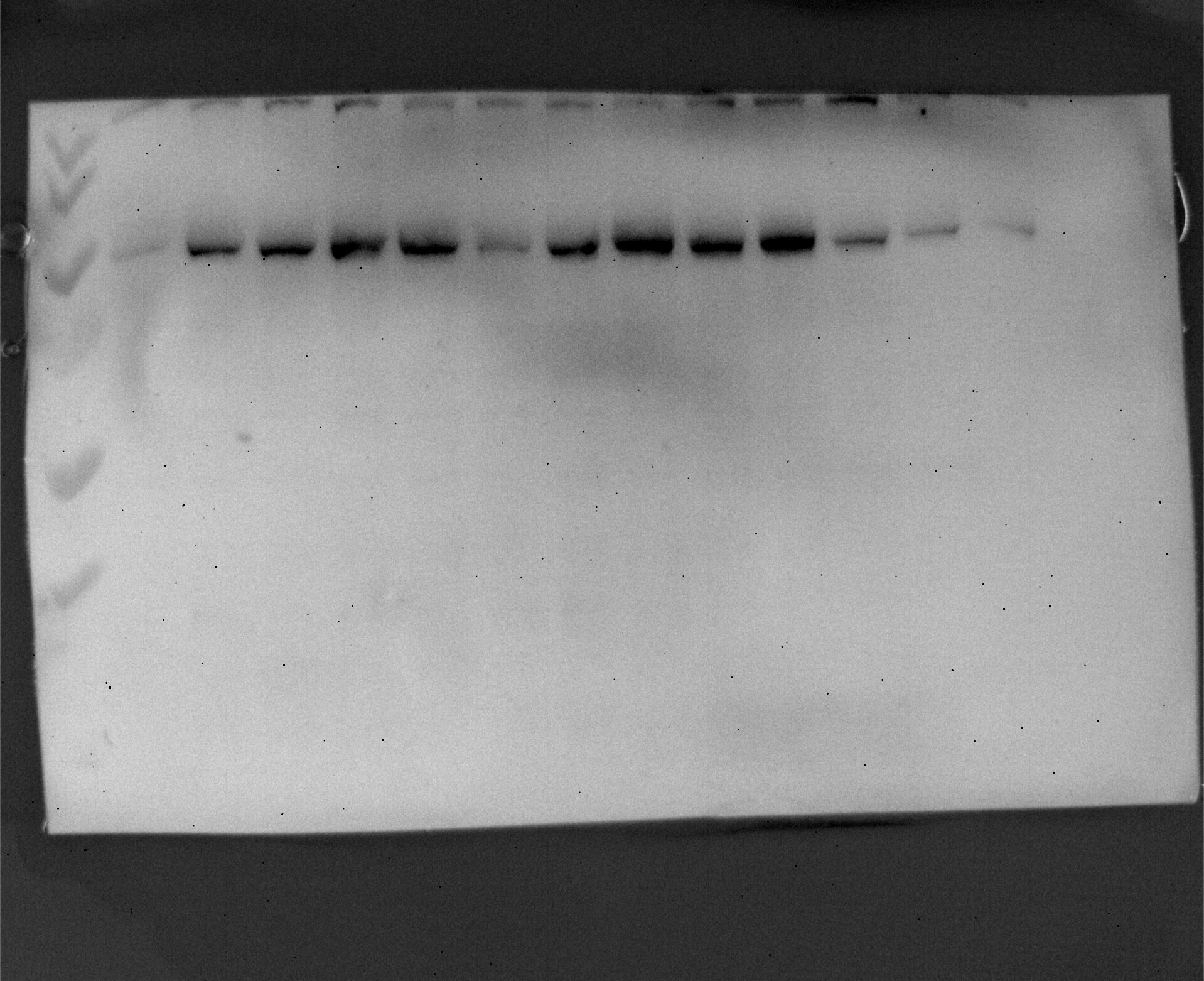

Supplement: Supplementary file 18 — EV Figure Source Data [file 44318_2025_370_MOESM18_ESM.zip › Figure EV1/Fig EV1B/pTBK1.jpg]

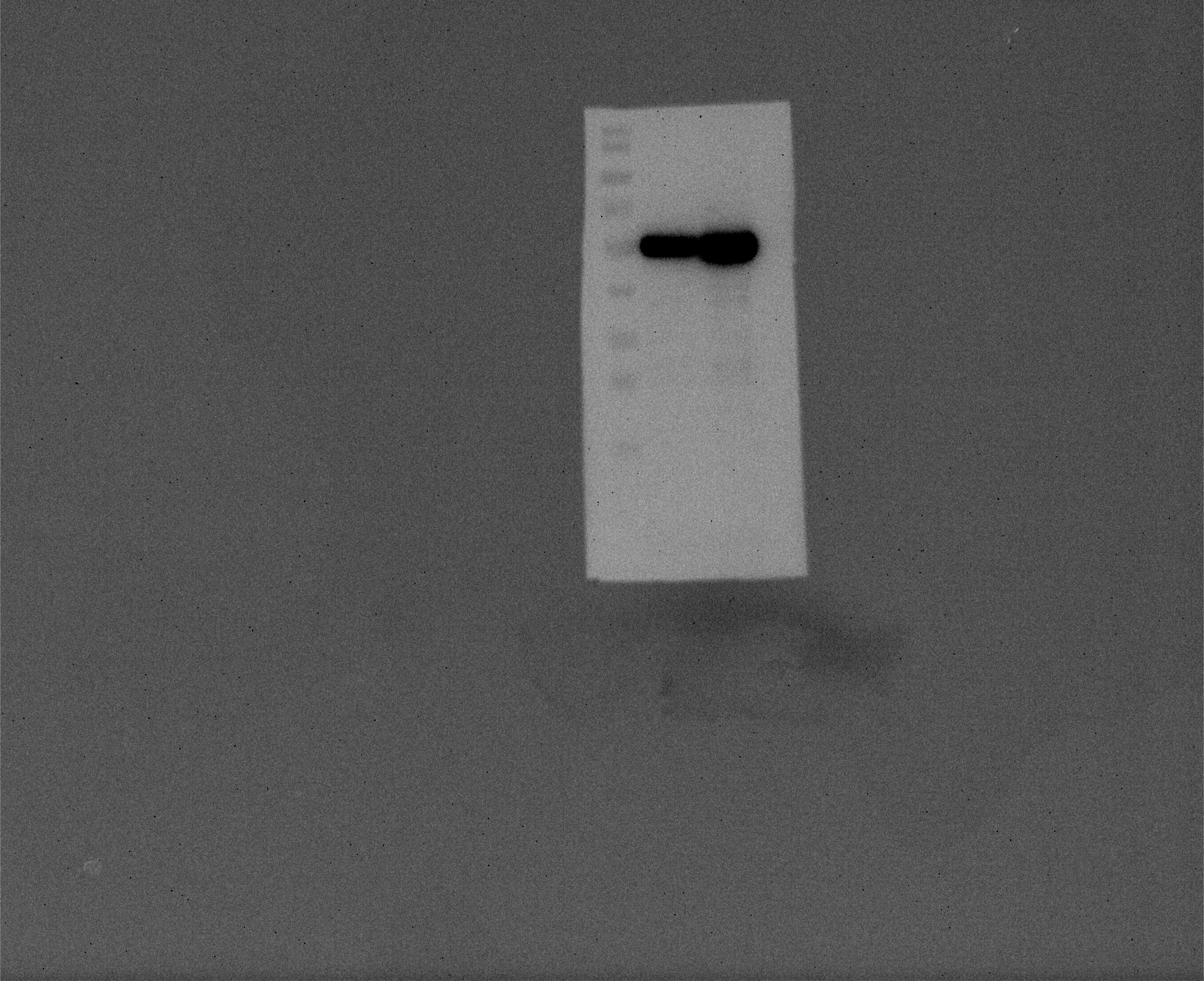

Supplement: Supplementary file 18 — EV Figure Source Data [file 44318_2025_370_MOESM18_ESM.zip › Figure EV1/Fig EV1H/IRF3.jpg]

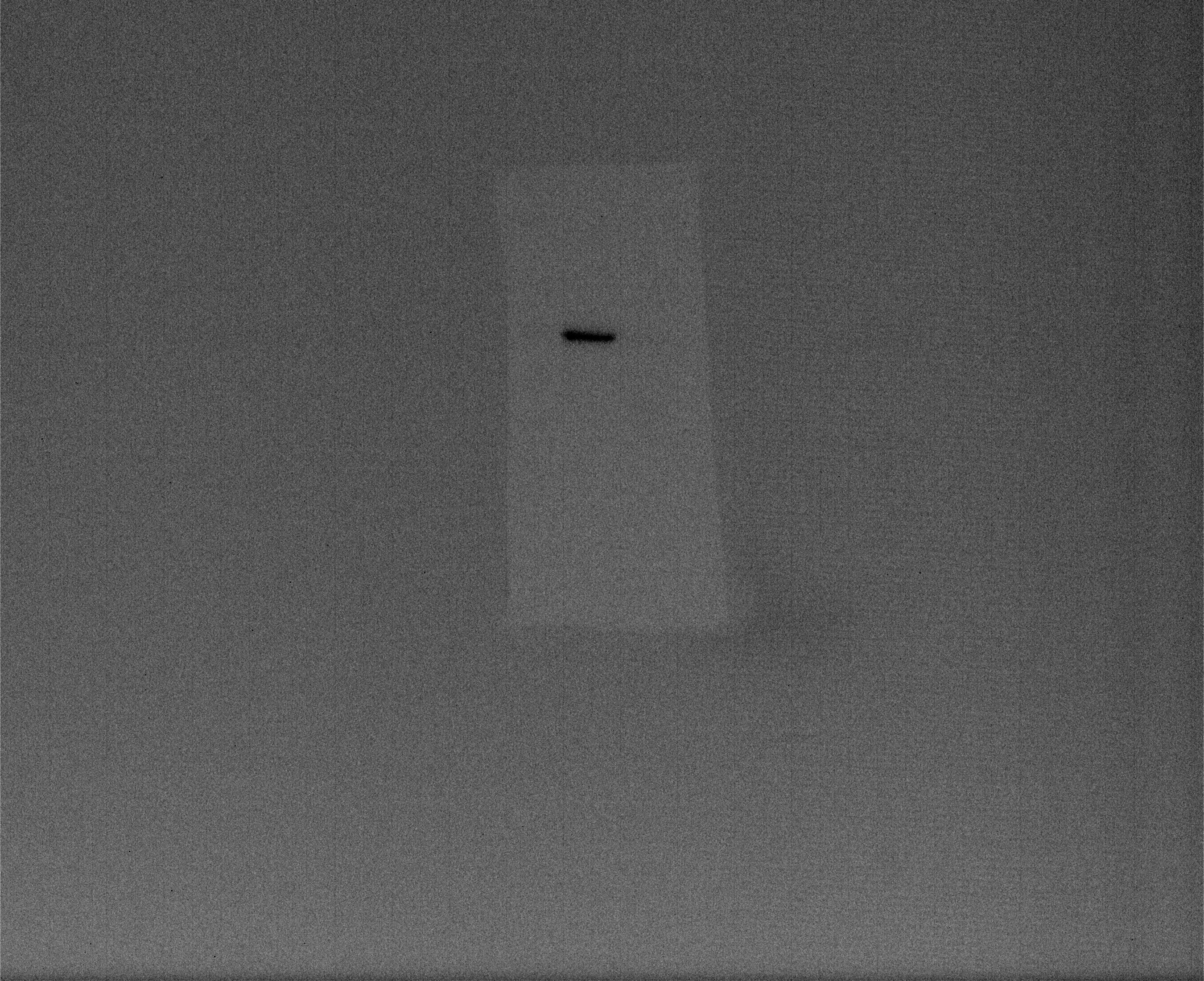

Supplement: Supplementary file 18 — EV Figure Source Data [file 44318_2025_370_MOESM18_ESM.zip › Figure EV1/Fig EV1H/STING.jpg]

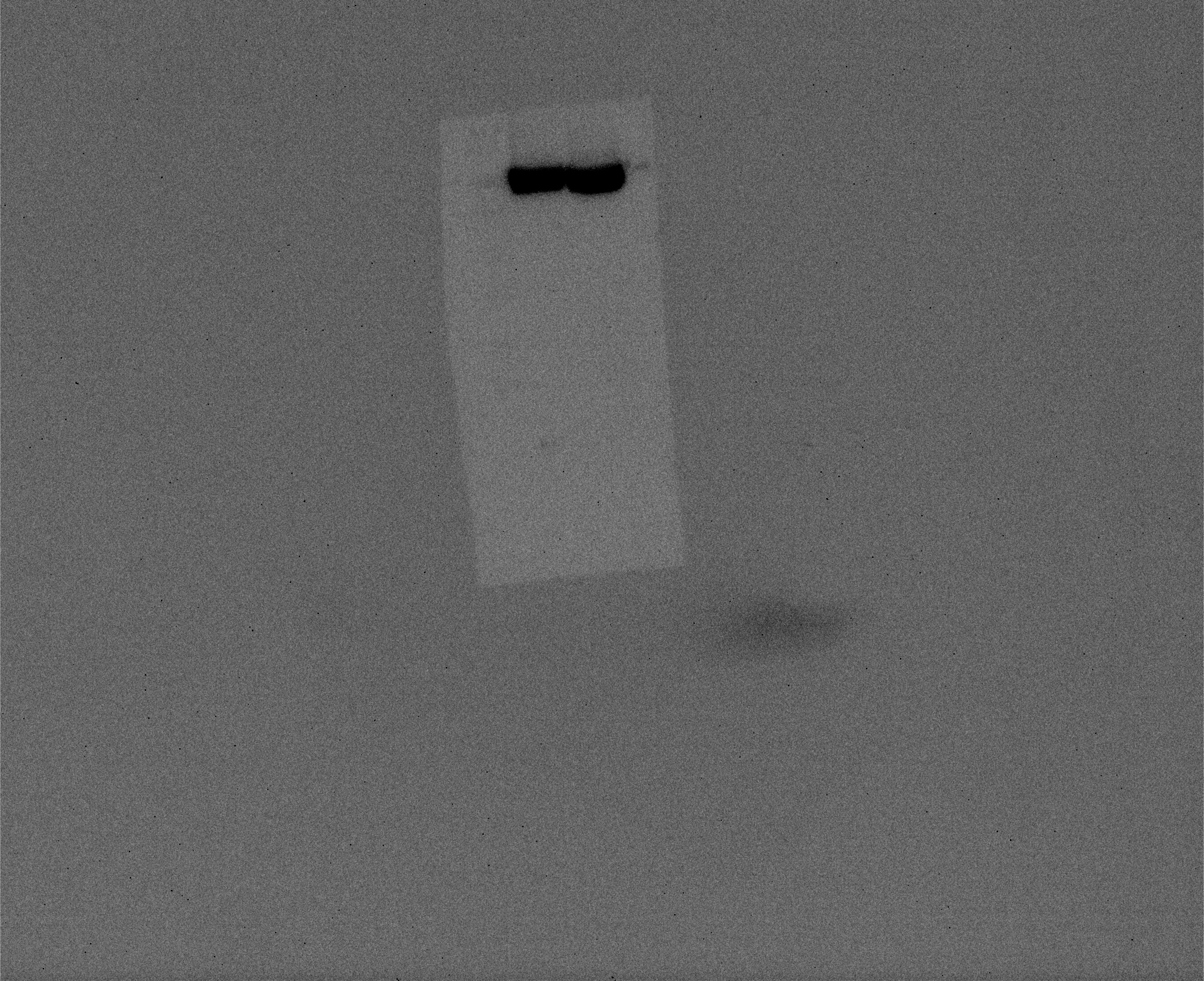

Supplement: Supplementary file 18 — EV Figure Source Data [file 44318_2025_370_MOESM18_ESM.zip › Figure EV1/Fig EV1H/TBK1.jpg]

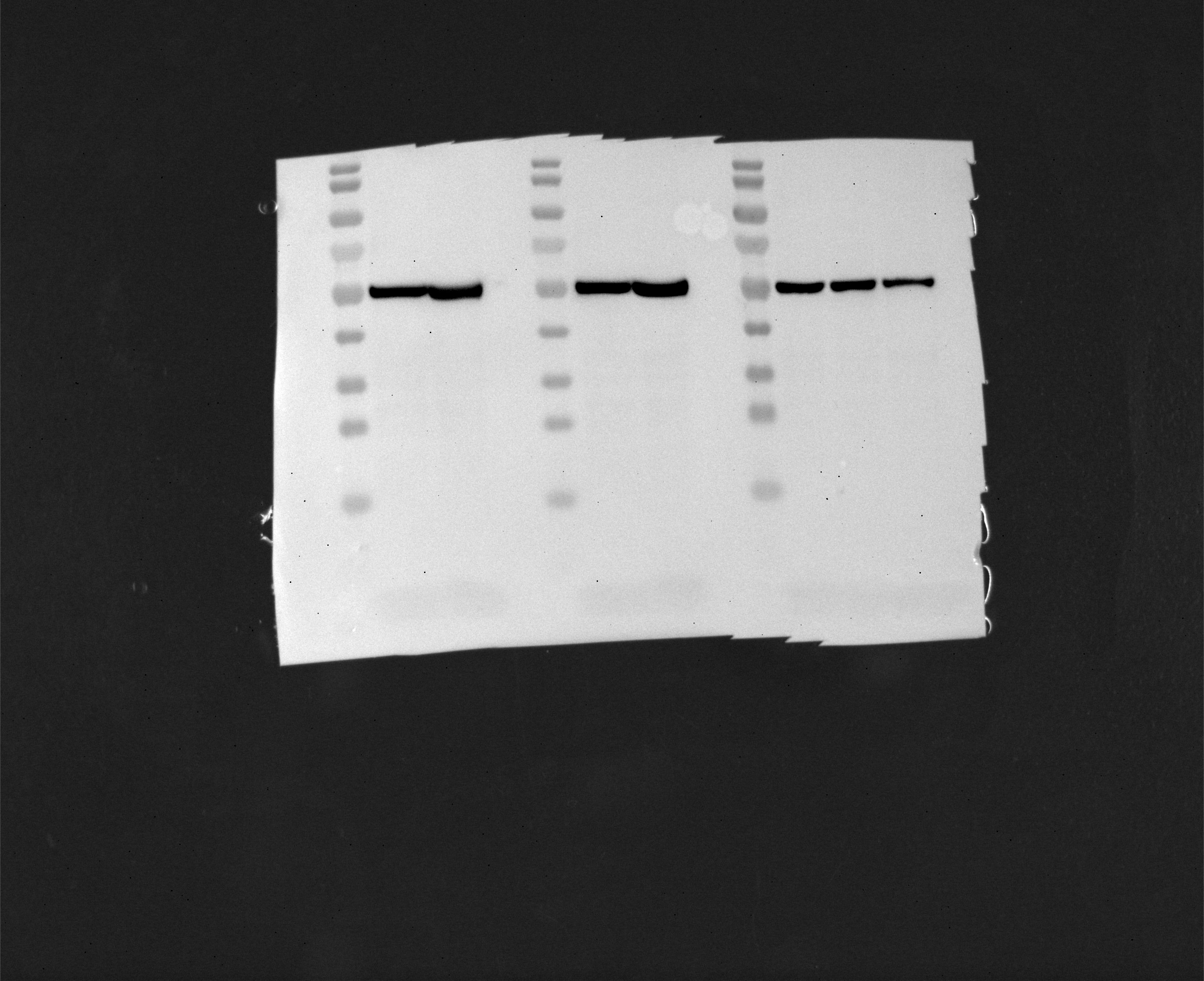

Supplement: Supplementary file 18 — EV Figure Source Data [file 44318_2025_370_MOESM18_ESM.zip › Figure EV1/Fig EV1H/a-tub.jpg]

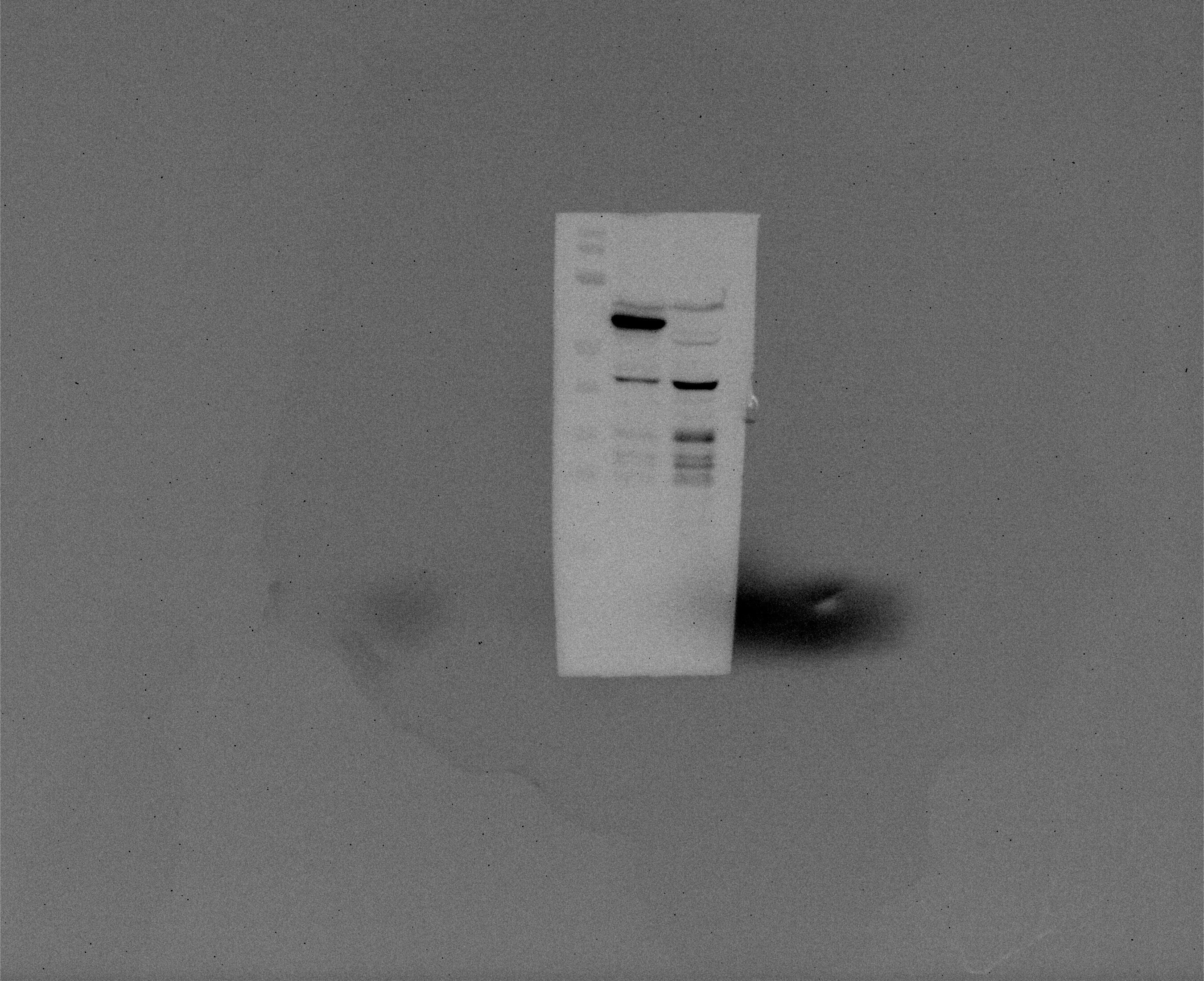

Supplement: Supplementary file 18 — EV Figure Source Data [file 44318_2025_370_MOESM18_ESM.zip › Figure EV1/Fig EV1H/cGAS.jpg]

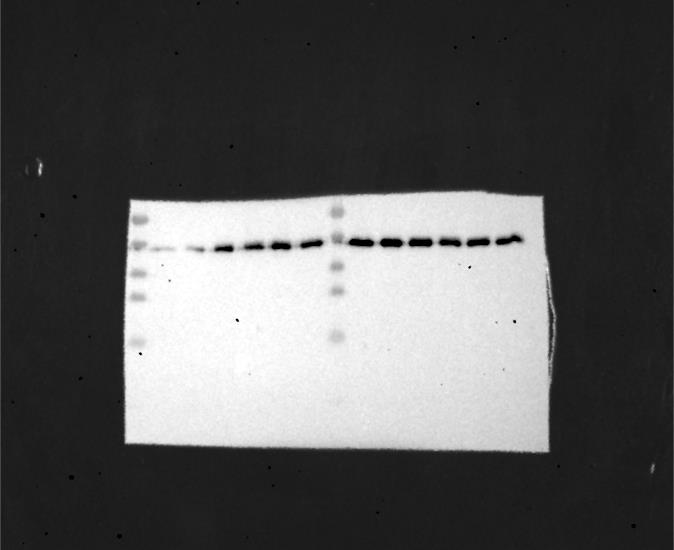

Supplement: Supplementary file 18 — EV Figure Source Data [file 44318_2025_370_MOESM18_ESM.zip › Figure EV2/Fig EV2C/STING.jpg]

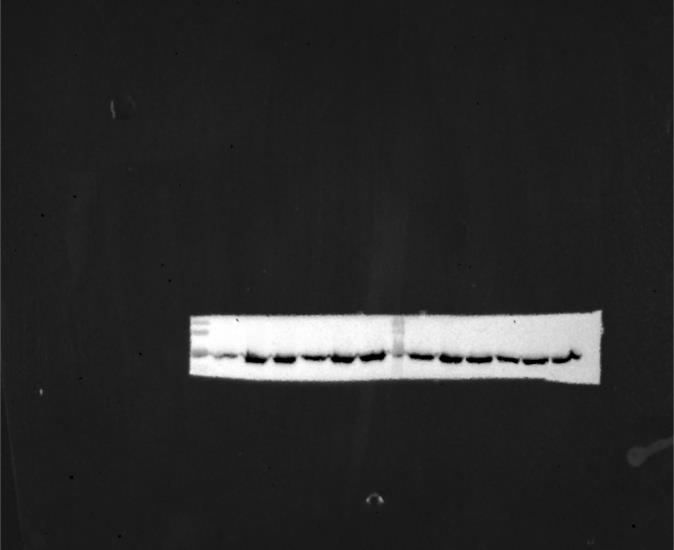

Supplement: Supplementary file 18 — EV Figure Source Data [file 44318_2025_370_MOESM18_ESM.zip › Figure EV2/Fig EV2C/TBK1.jpg]

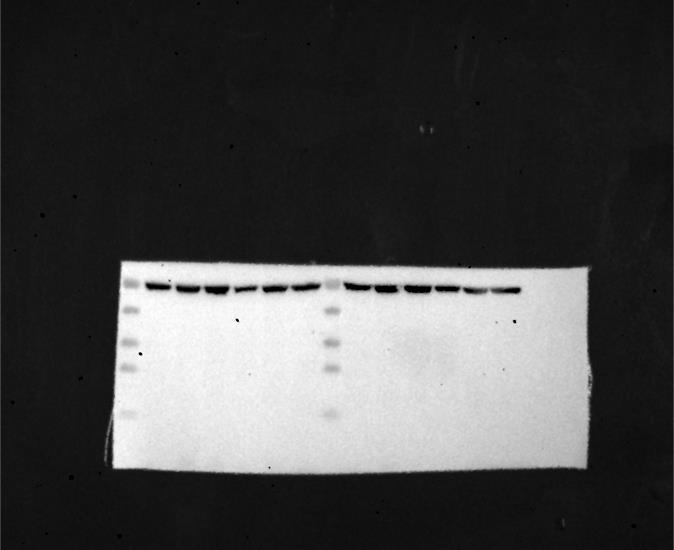

Supplement: Supplementary file 18 — EV Figure Source Data [file 44318_2025_370_MOESM18_ESM.zip › Figure EV2/Fig EV2C/a-tub.jpg]

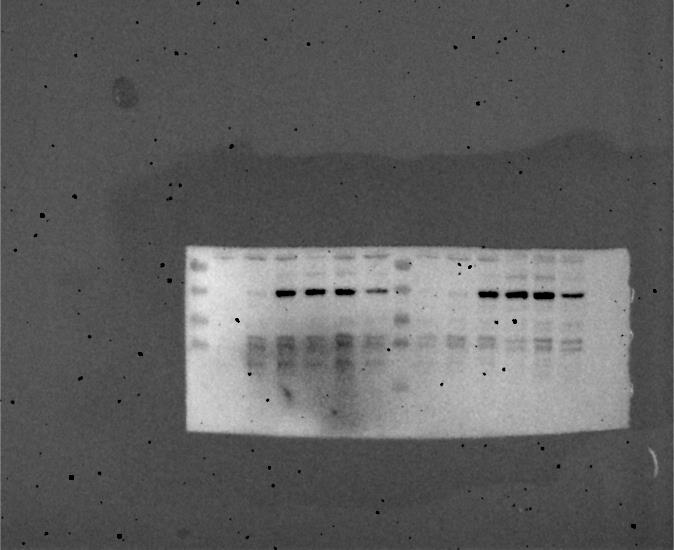

Supplement: Supplementary file 18 — EV Figure Source Data [file 44318_2025_370_MOESM18_ESM.zip › Figure EV2/Fig EV2C/pSTING.jpg]

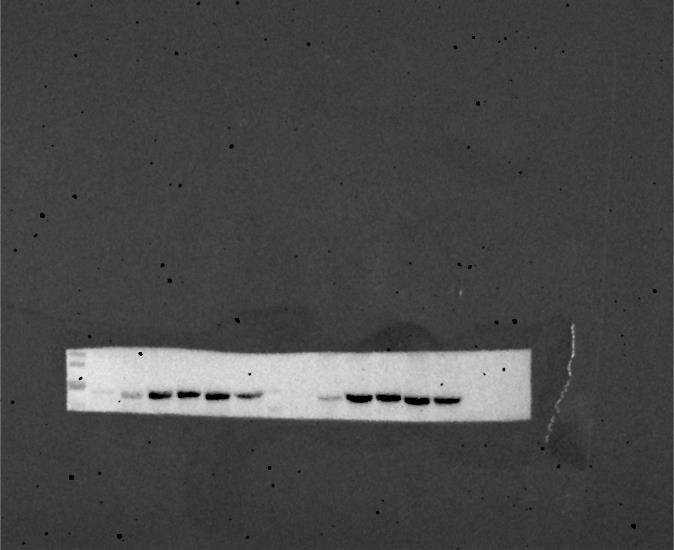

Supplement: Supplementary file 18 — EV Figure Source Data [file 44318_2025_370_MOESM18_ESM.zip › Figure EV2/Fig EV2C/pTBK1.jpg]

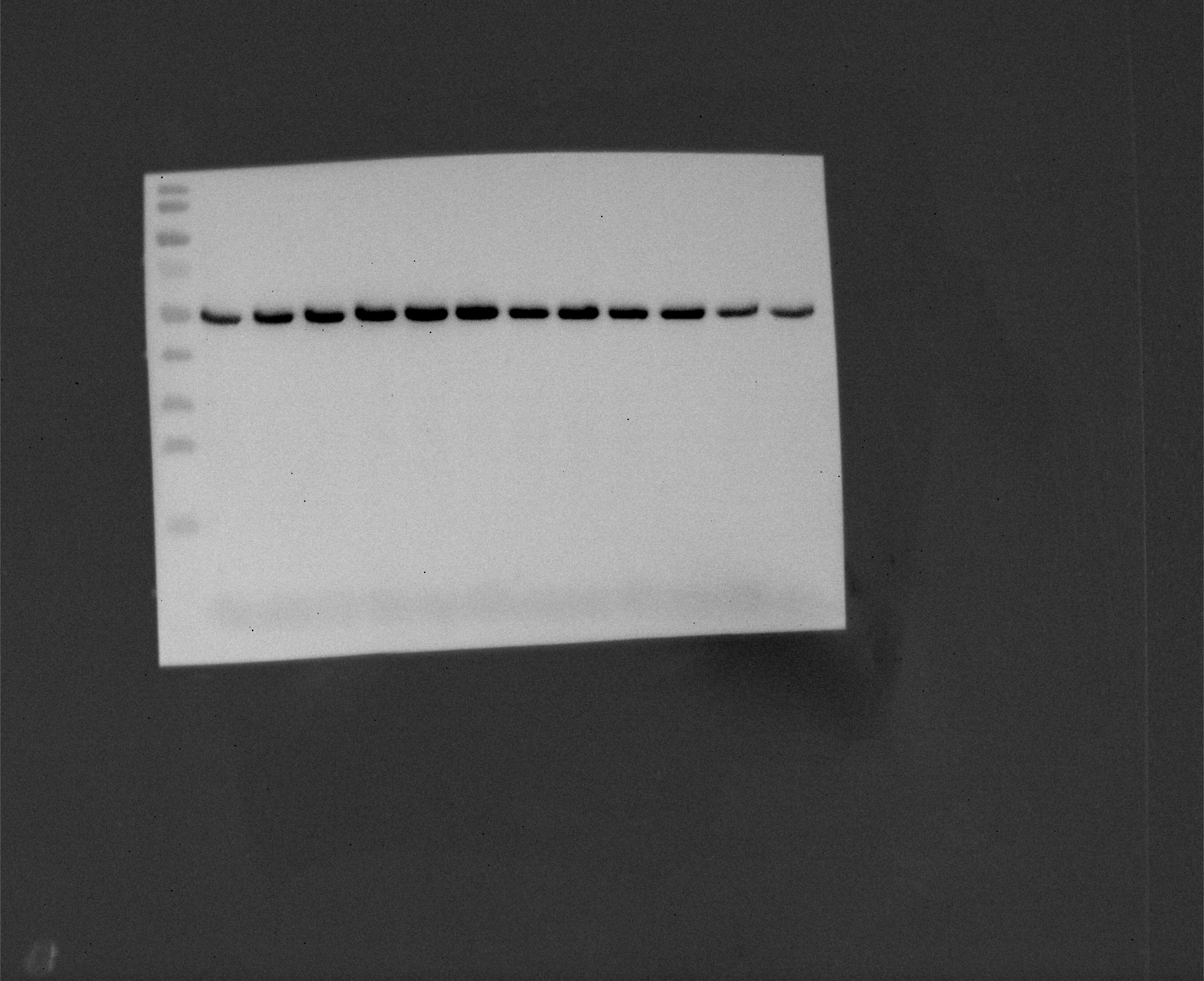

Supplement: Supplementary file 18 — EV Figure Source Data [file 44318_2025_370_MOESM18_ESM.zip › Figure EV2/Fig EV2F/IRF3.jpg]

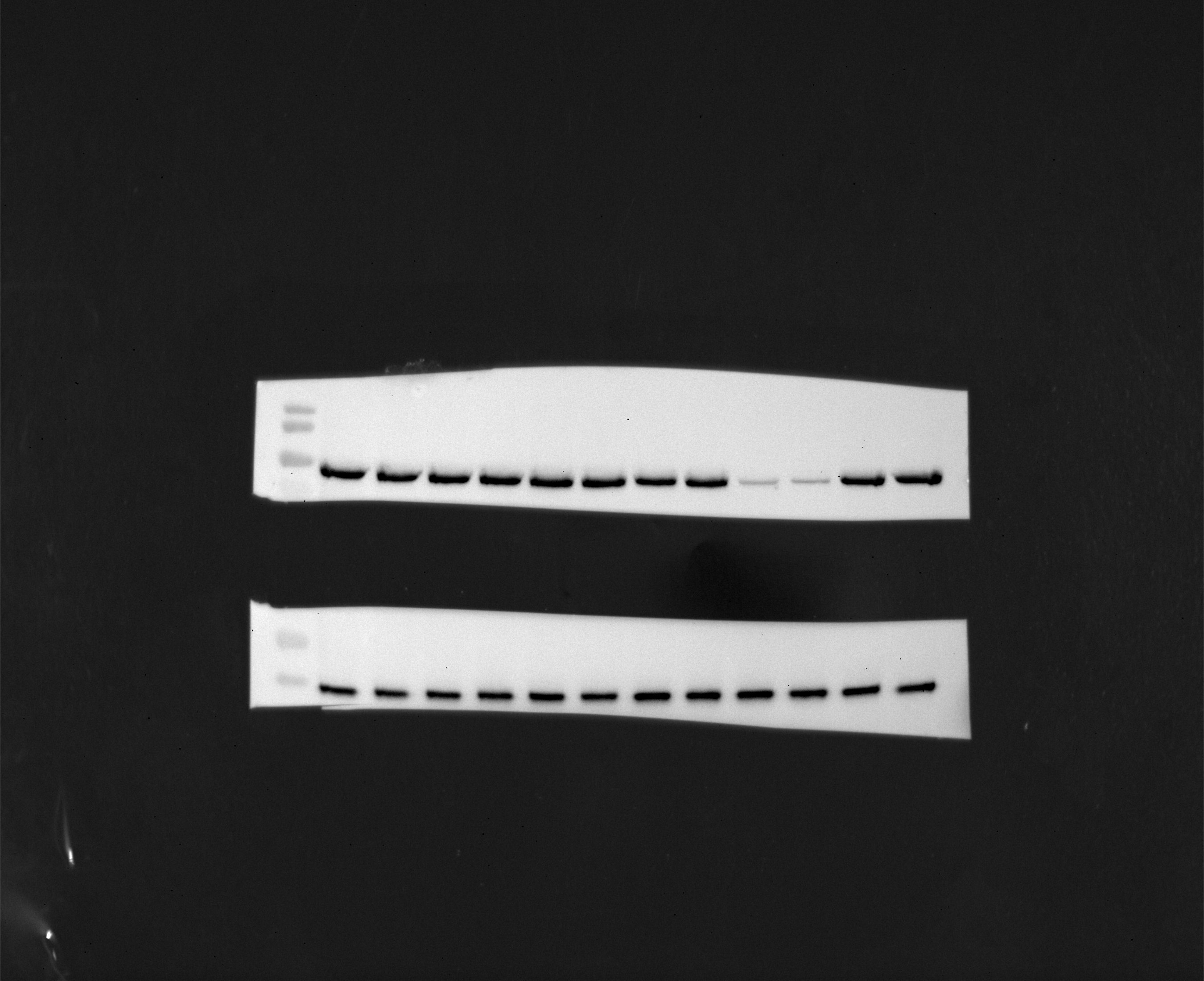

Supplement: Supplementary file 18 — EV Figure Source Data [file 44318_2025_370_MOESM18_ESM.zip › Figure EV2/Fig EV2F/TBK1 and STING.jpg]

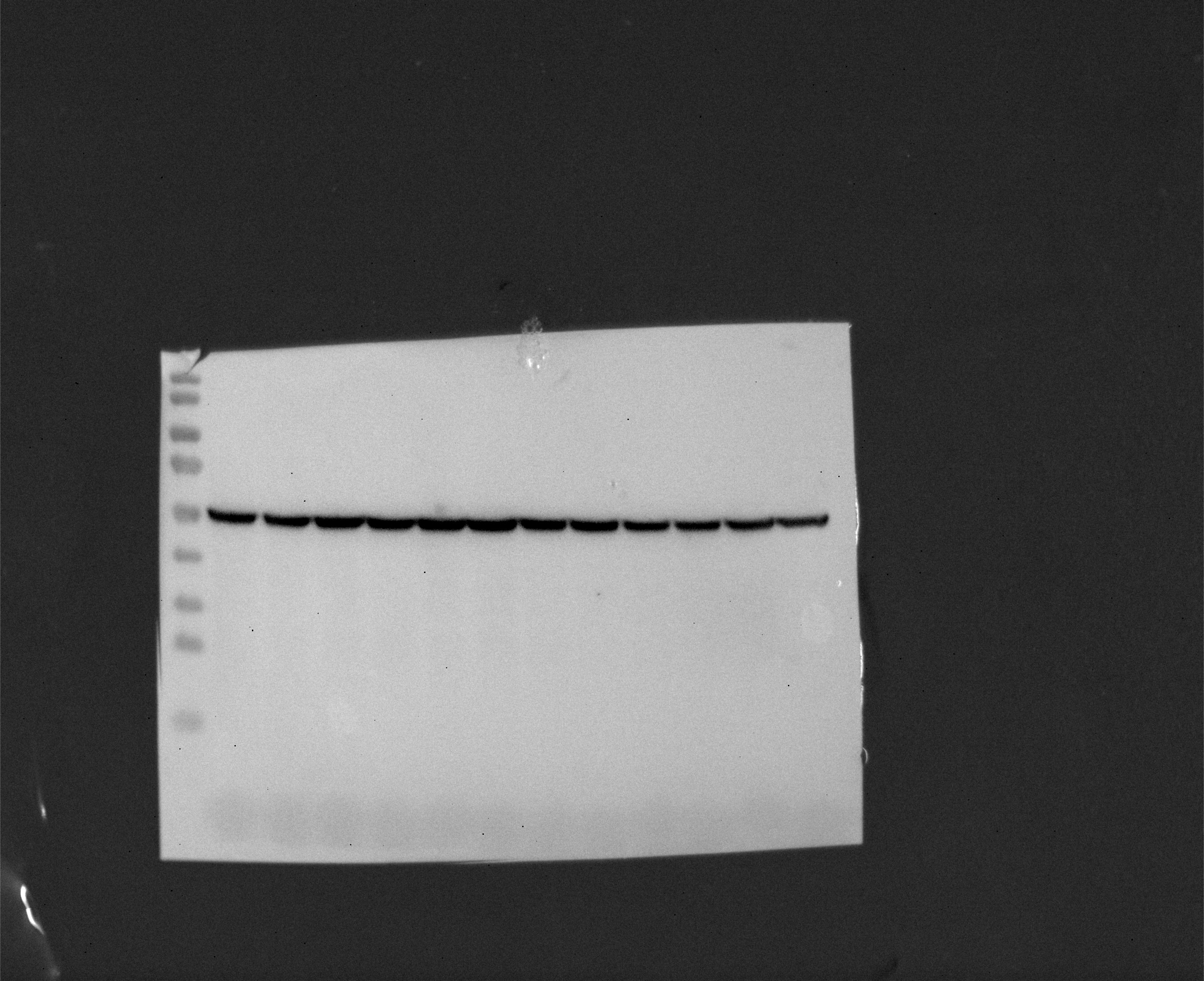

Supplement: Supplementary file 18 — EV Figure Source Data [file 44318_2025_370_MOESM18_ESM.zip › Figure EV2/Fig EV2F/a-tub.jpg]

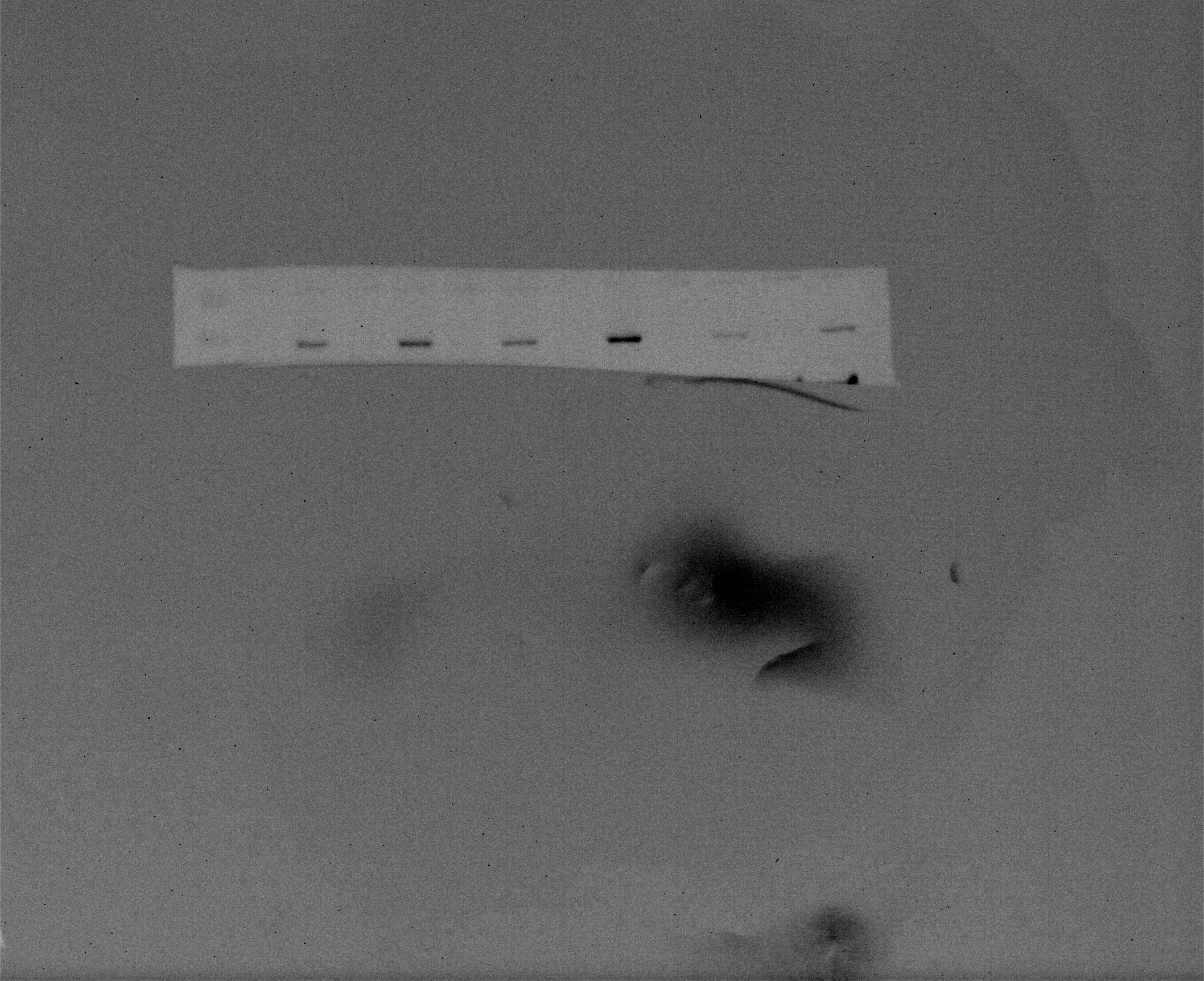

Supplement: Supplementary file 18 — EV Figure Source Data [file 44318_2025_370_MOESM18_ESM.zip › Figure EV2/Fig EV2F/pSTING.jpg]

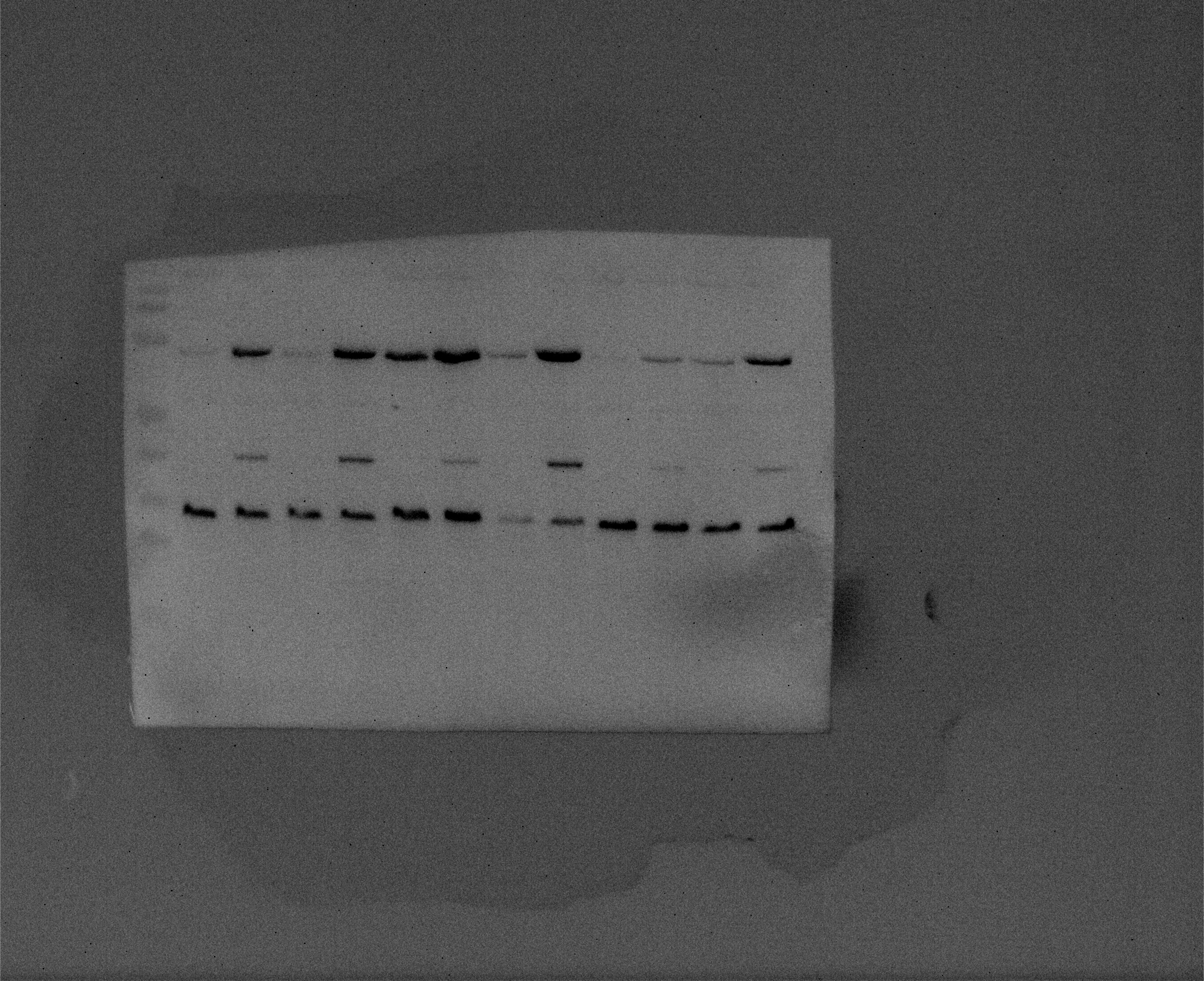

Supplement: Supplementary file 18 — EV Figure Source Data [file 44318_2025_370_MOESM18_ESM.zip › Figure EV2/Fig EV2F/pTBK1 and pSTING.jpg]

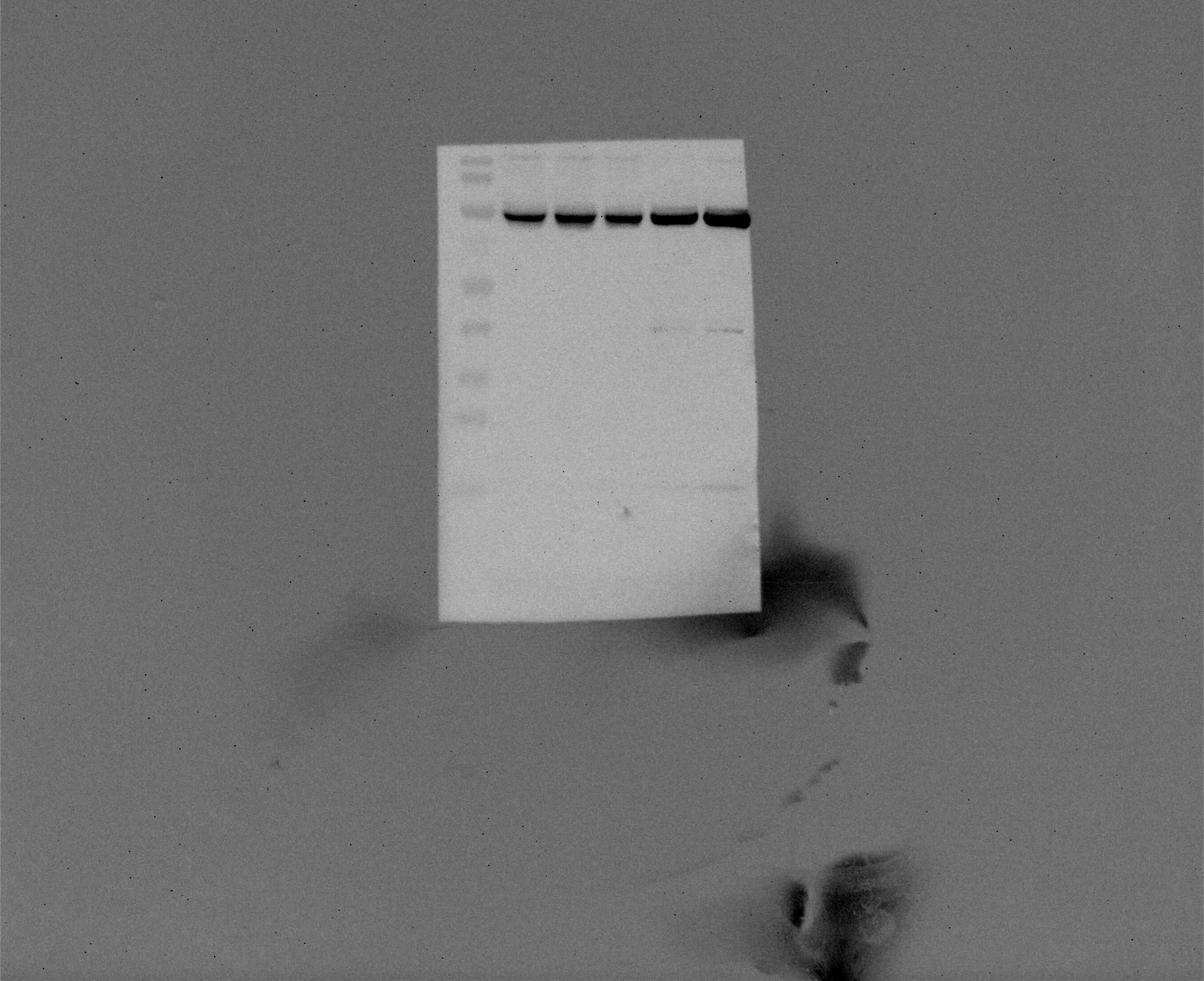

Supplement: Supplementary file 18 — EV Figure Source Data [file 44318_2025_370_MOESM18_ESM.zip › Figure EV2/Fig EV2K/TBK1.jpg]

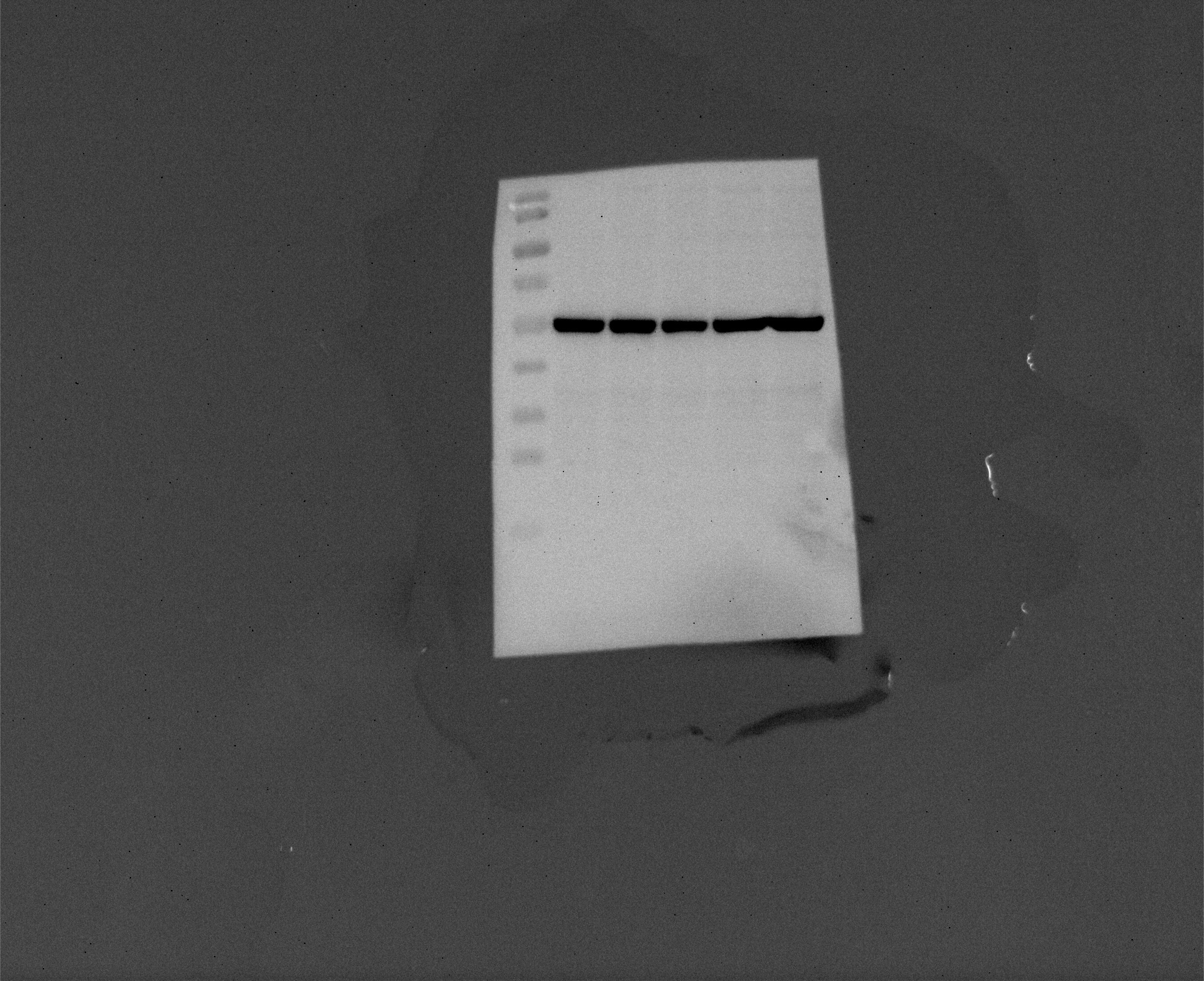

Supplement: Supplementary file 18 — EV Figure Source Data [file 44318_2025_370_MOESM18_ESM.zip › Figure EV2/Fig EV2K/alpha tub.jpg]

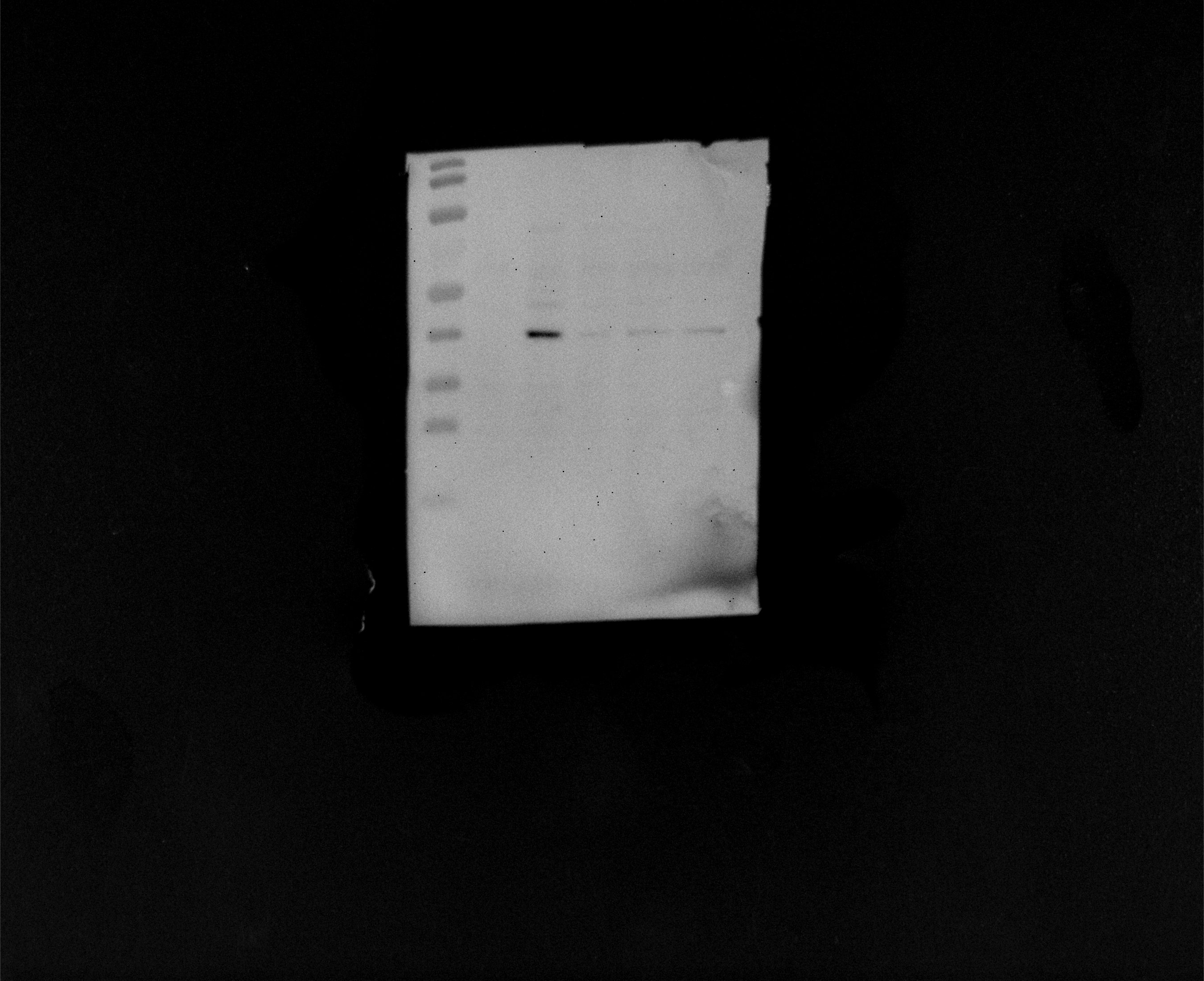

Supplement: Supplementary file 18 — EV Figure Source Data [file 44318_2025_370_MOESM18_ESM.zip › Figure EV2/Fig EV2K/pSTING.jpg]

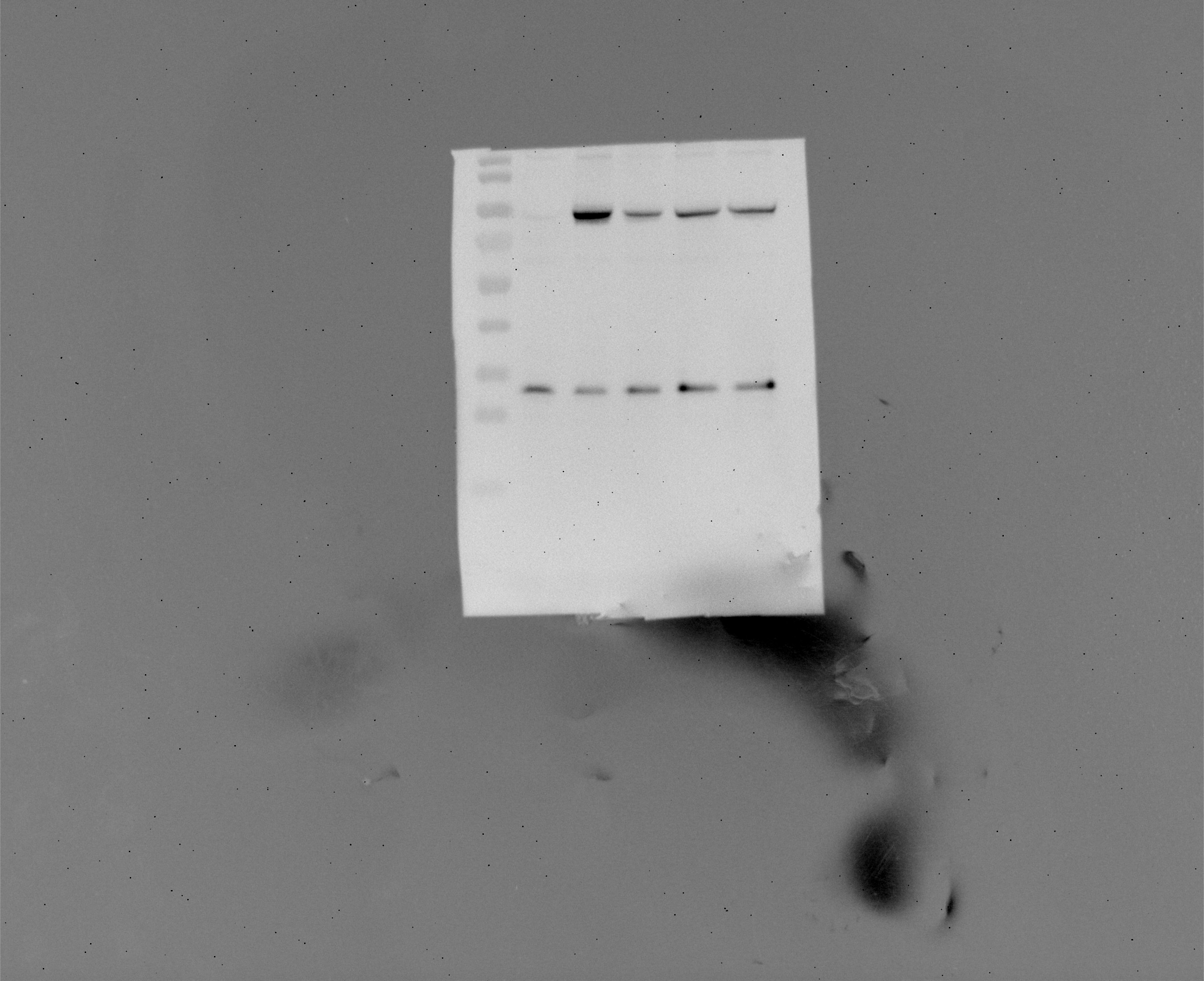

Supplement: Supplementary file 18 — EV Figure Source Data [file 44318_2025_370_MOESM18_ESM.zip › Figure EV2/Fig EV2K/pTBK1.jpg]

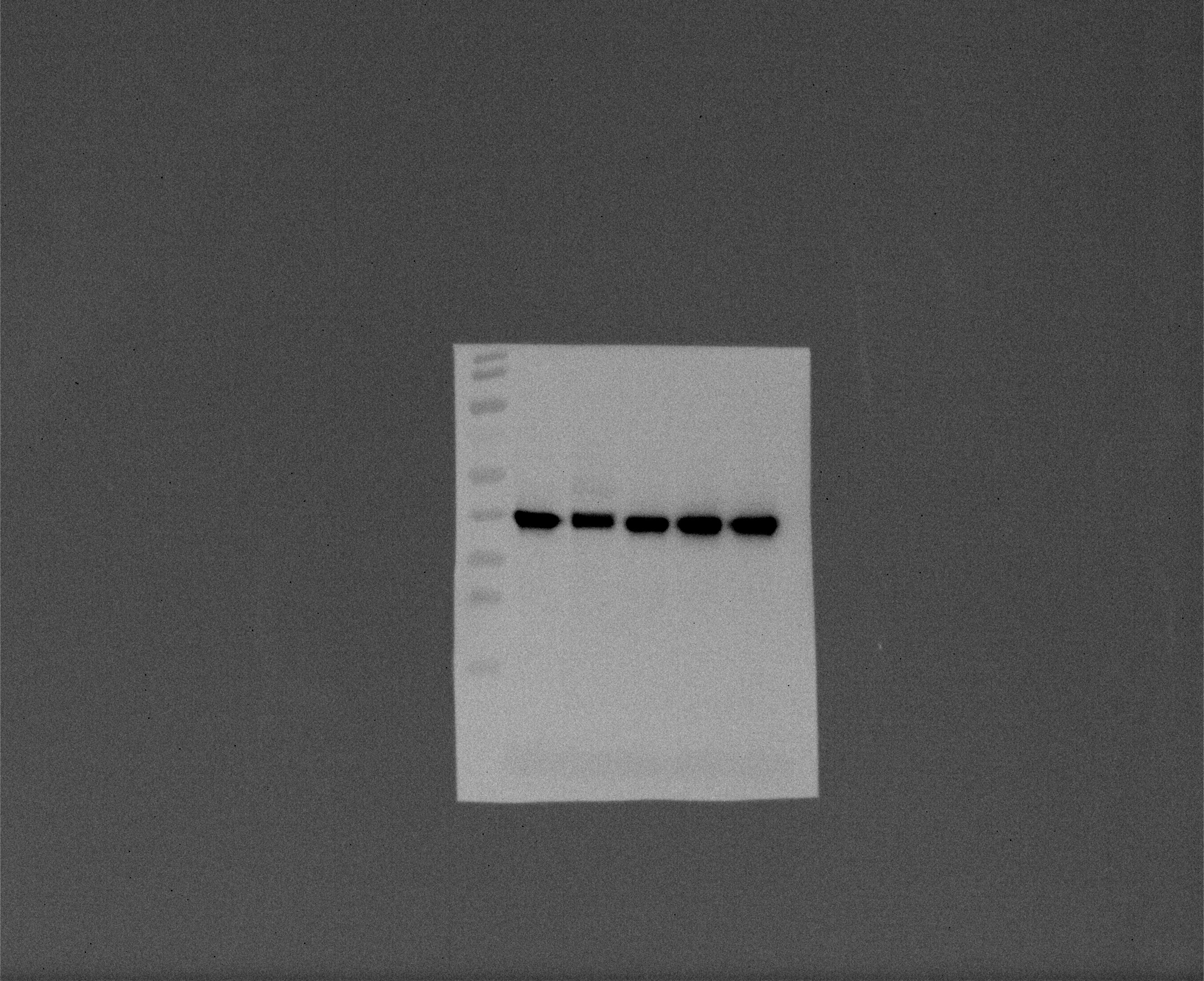

Supplement: Supplementary file 18 — EV Figure Source Data [file 44318_2025_370_MOESM18_ESM.zip › Figure EV4/Fig EV4H/STING.jpg]

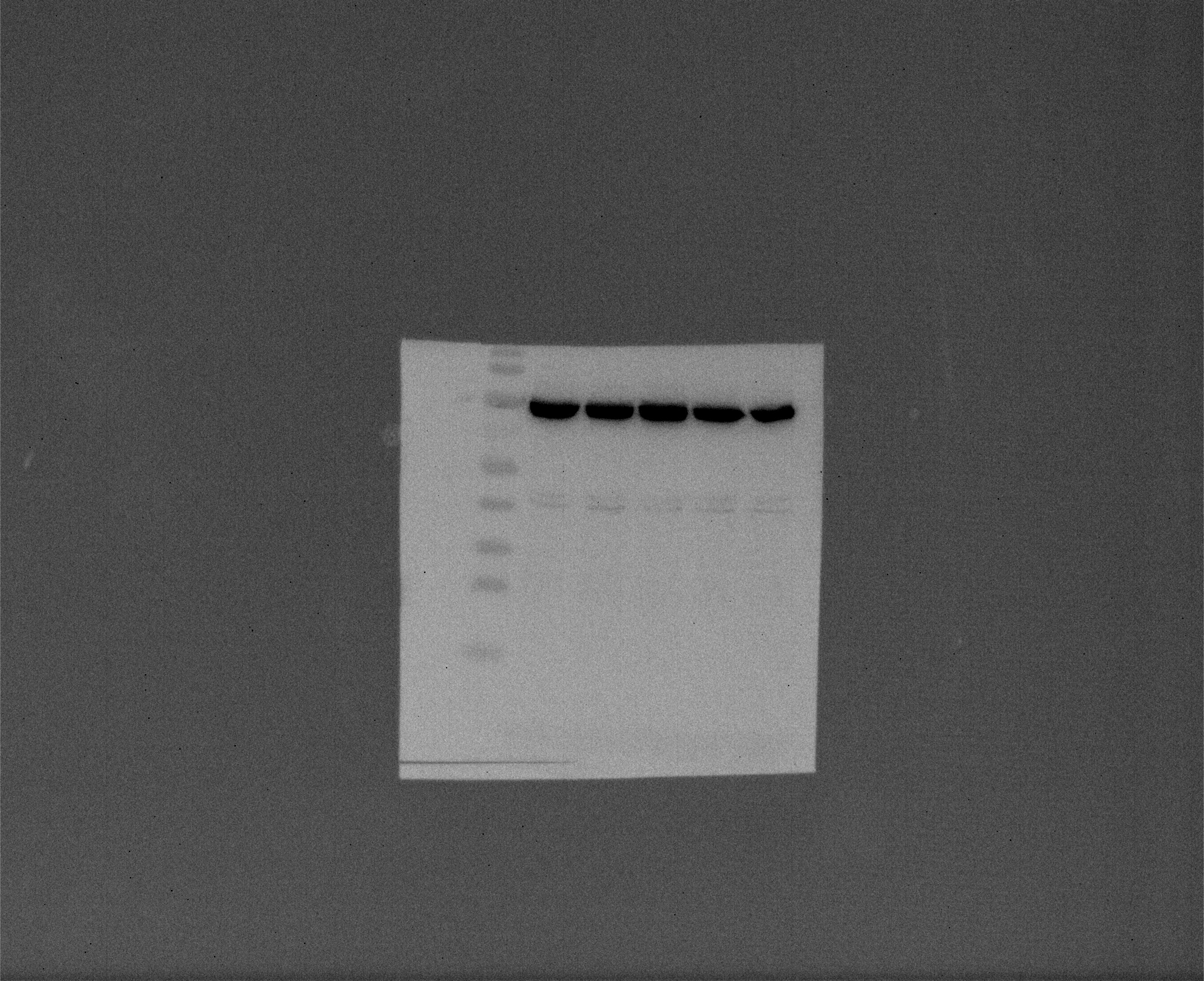

Supplement: Supplementary file 18 — EV Figure Source Data [file 44318_2025_370_MOESM18_ESM.zip › Figure EV4/Fig EV4H/TBK1.jpg]

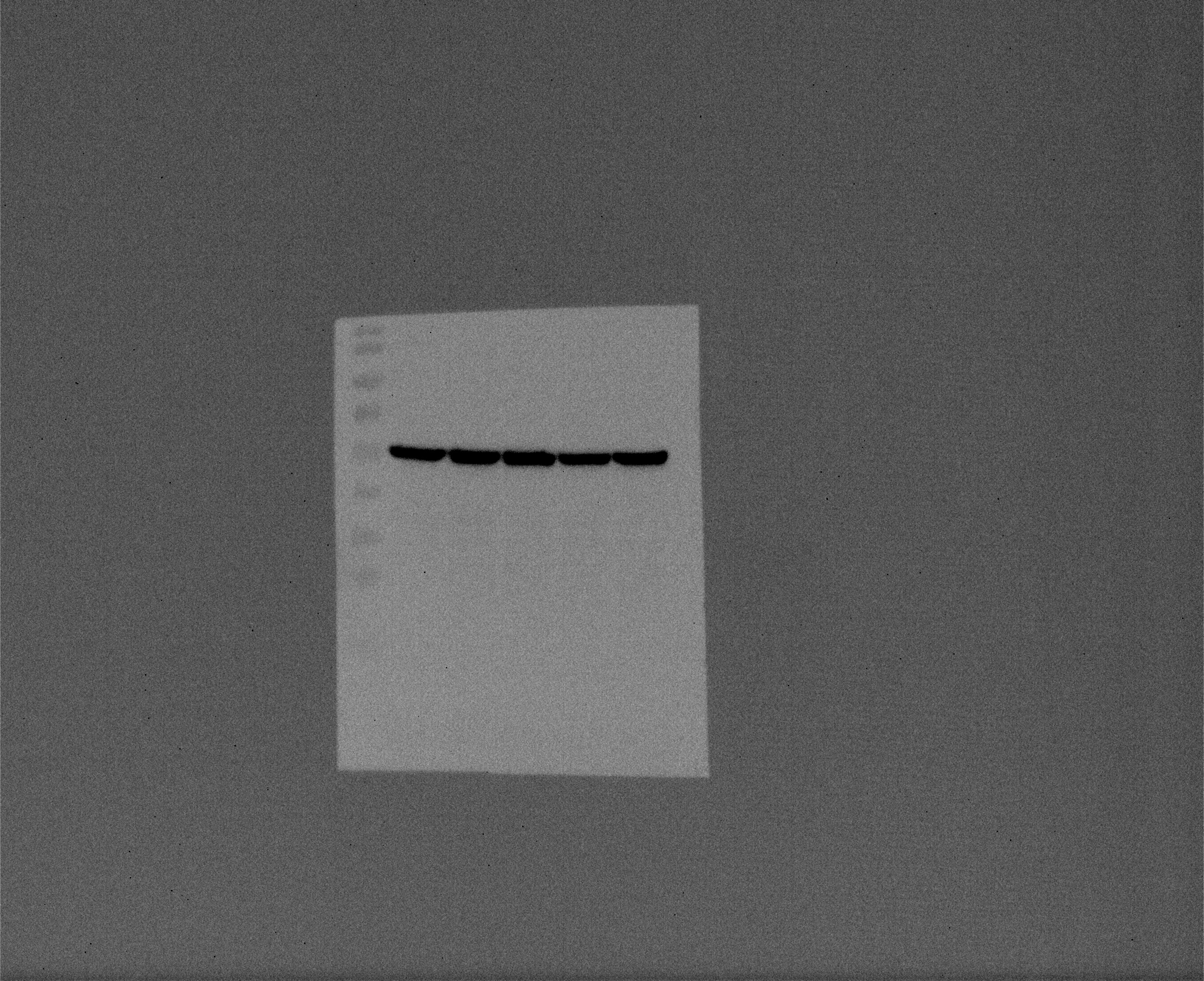

Supplement: Supplementary file 18 — EV Figure Source Data [file 44318_2025_370_MOESM18_ESM.zip › Figure EV4/Fig EV4H/a-tub.jpg]

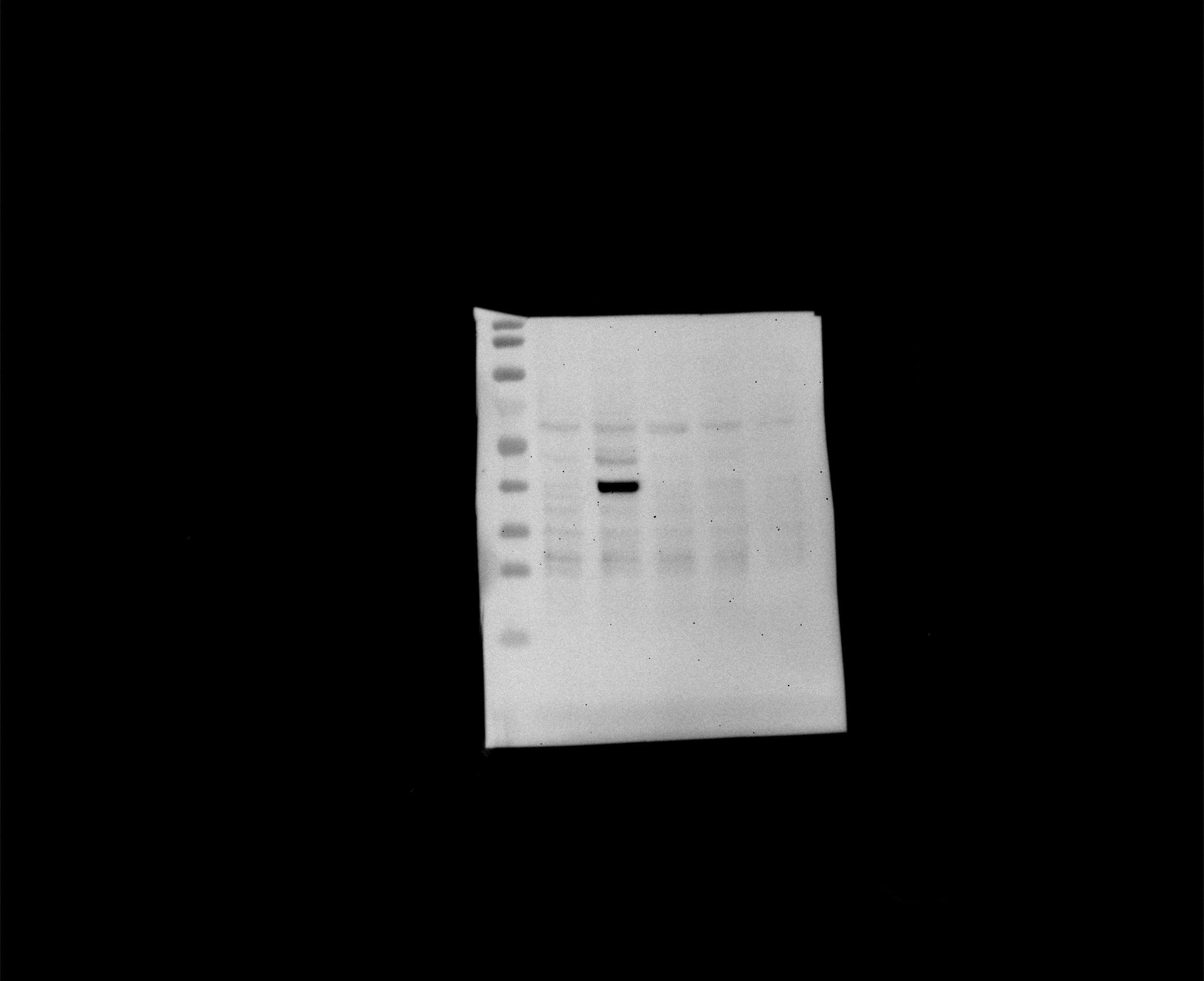

Supplement: Supplementary file 18 — EV Figure Source Data [file 44318_2025_370_MOESM18_ESM.zip › Figure EV4/Fig EV4H/pSTING.jpg]

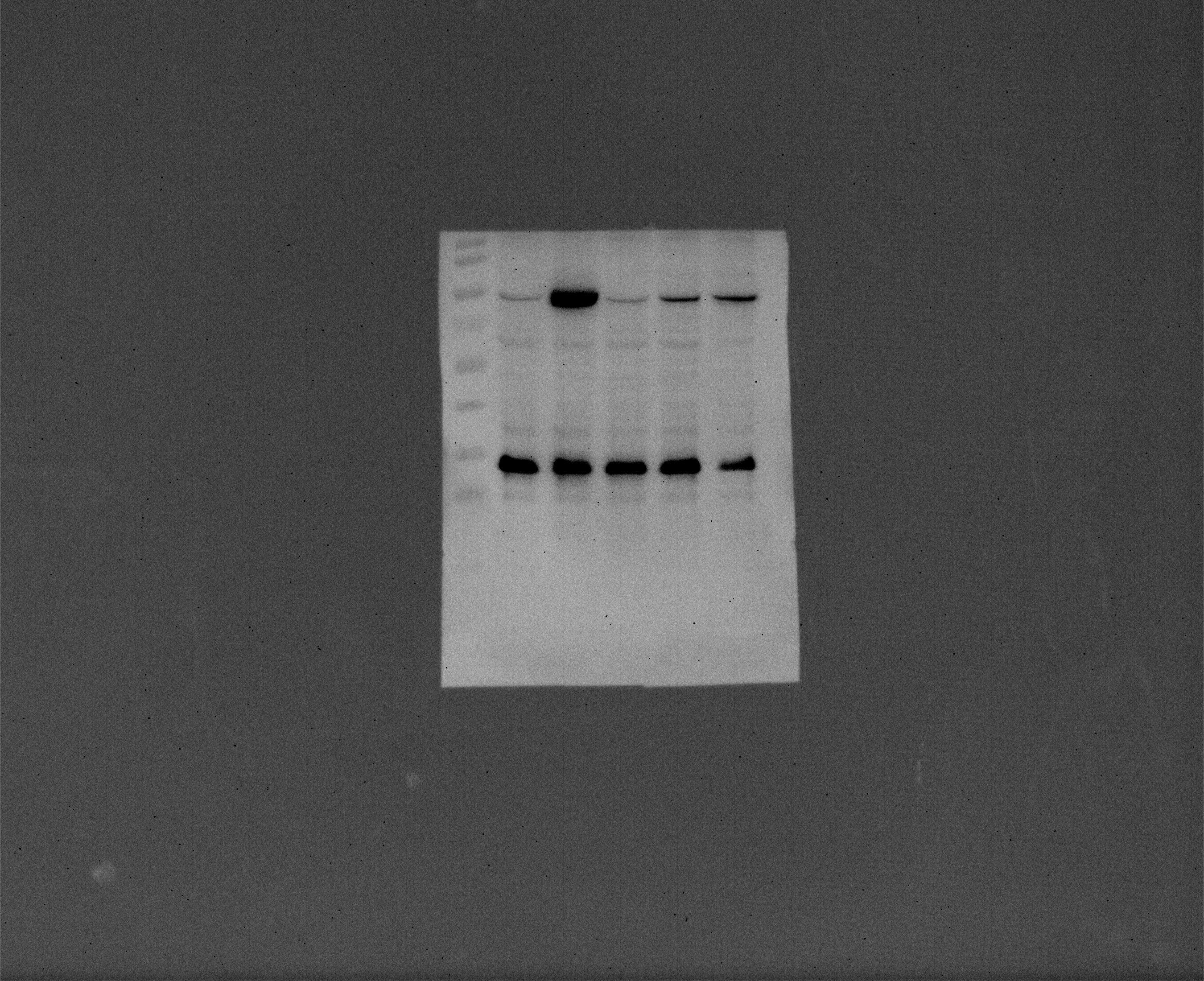

Supplement: Supplementary file 18 — EV Figure Source Data [file 44318_2025_370_MOESM18_ESM.zip › Figure EV4/Fig EV4H/pTBK1.jpg]

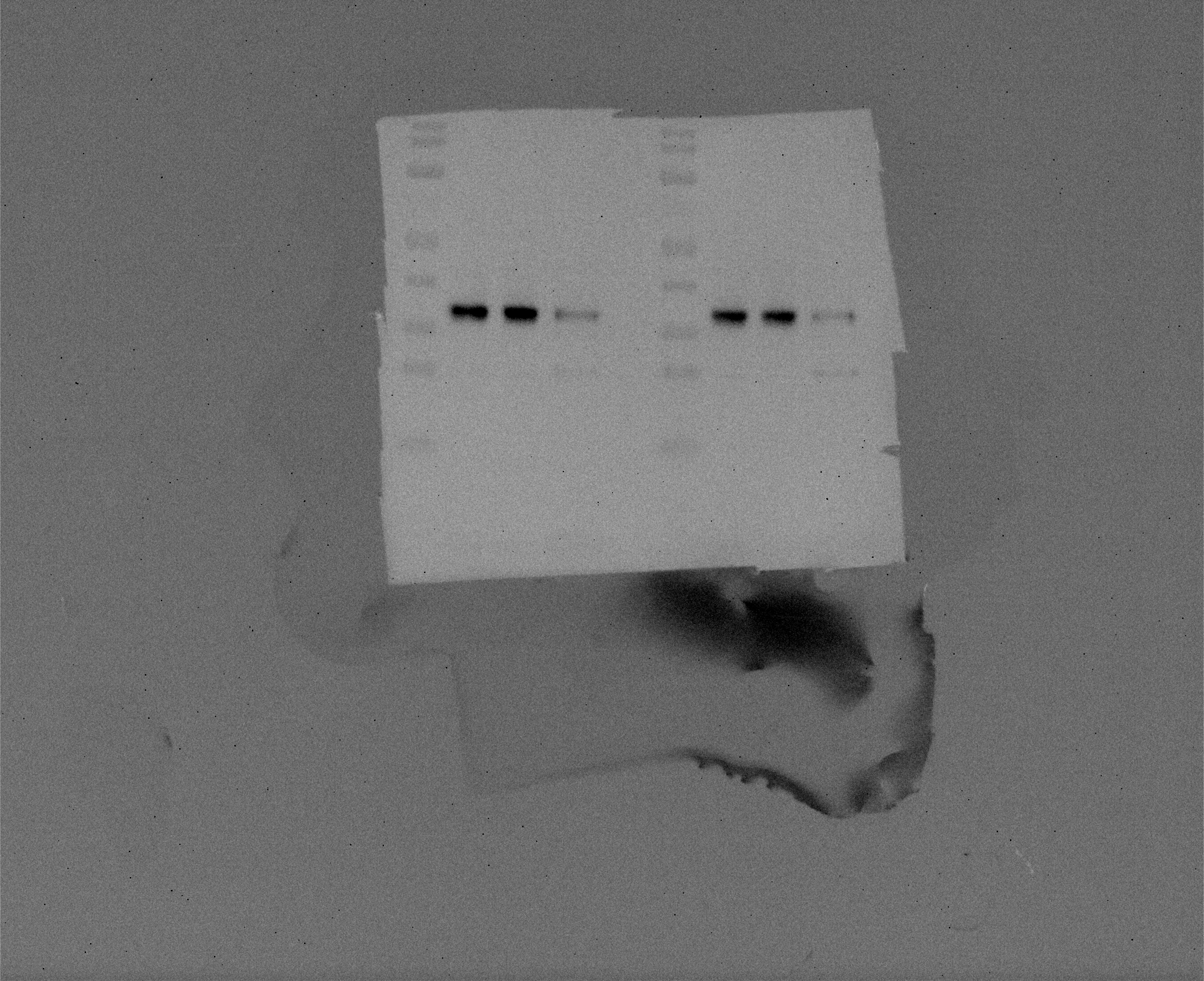

Supplement: Supplementary file 18 — EV Figure Source Data [file 44318_2025_370_MOESM18_ESM.zip › Figure EV5/Fig EV5D/TREX1.jpg]

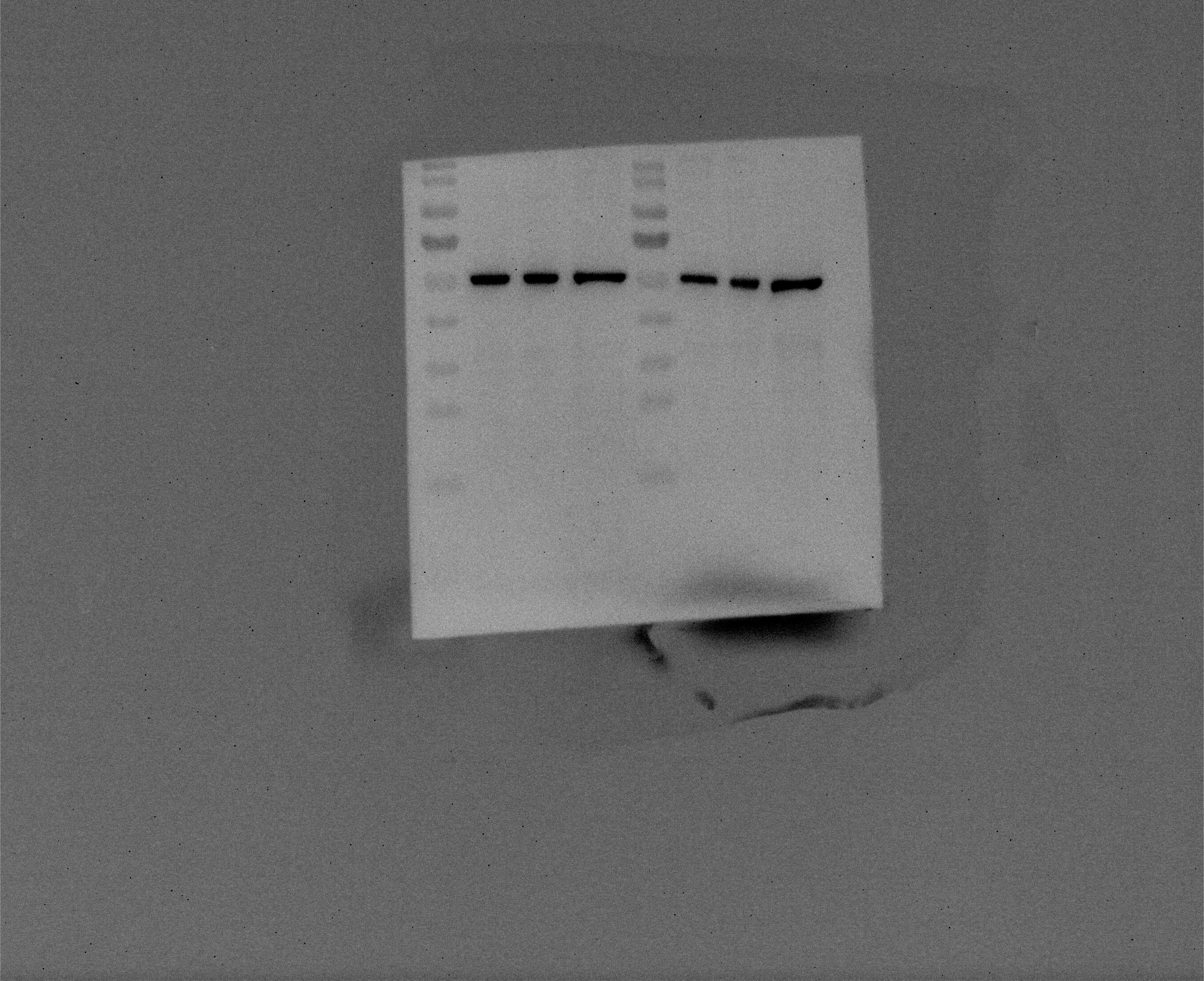

Supplement: Supplementary file 18 — EV Figure Source Data [file 44318_2025_370_MOESM18_ESM.zip › Figure EV5/Fig EV5D/a-tub.jpg]
