# Supplementary material for: A novel biosensor for the spatiotemporal analysis of STING activation during innate immune responses to dsDNA
Source: EMBO J. 2025 Feb 21;44(7):2157–82. doi: 10.1038/s44318-025-00370-y (PMC11962129; doi:10.1038/s44318-025-00370-y)
Supplement: Supplementary file 19 — Expanded View Figures [file 44318_2025_370_MOESM19_ESM.pdf]

Expanded View Figures

Figure EV1. STING signalling analyses in HEK293T, HFF1 and HeLa cells.

(**A**) PiggyBAC construct containing the SIRF biosensor. (**B**) Western blots of wt and SIRF biosensor HeLa cells treated as indicated. Note that the samples of HeLa biosensor cells correspond to the same experiment shown in Fig. 1C. (**C**) qRT-PCR analyses of wt and SIRF biosensor HeLa cells treated as indicated and represent as mean ± SD of 7–8 biological replicates. (**D**, **E**) Immunofluorescence analyses of wt HeLa cells treated as indicated. (**F**) Live cell confocal imaging analyses of HEK293T and HFF-1 cGAMP-biosensor cells treated with 2 µM diABZI. Data in the mean ± SEM of relative fluorescence (compared to t = 0) of 20 randomly selected cells per condition. (**G**) IFNb-Luc reporter assays in HEK293T cells transfected with empty plasmid or wt STING, or HEK293T cGAMP-biosensor cells, and treated with the indicated concentrations of the STING agonist diABZI, and represent as mean ± SD of 4 biological replicates. Note that HEK293T do not express endogenous cGAS or STING as shown in (**H**). (**H**) Western blots of wt HeK293T and HeLa cells. Scale bars = 10 µm. Source data are available online for this figure.

© The Author(s)

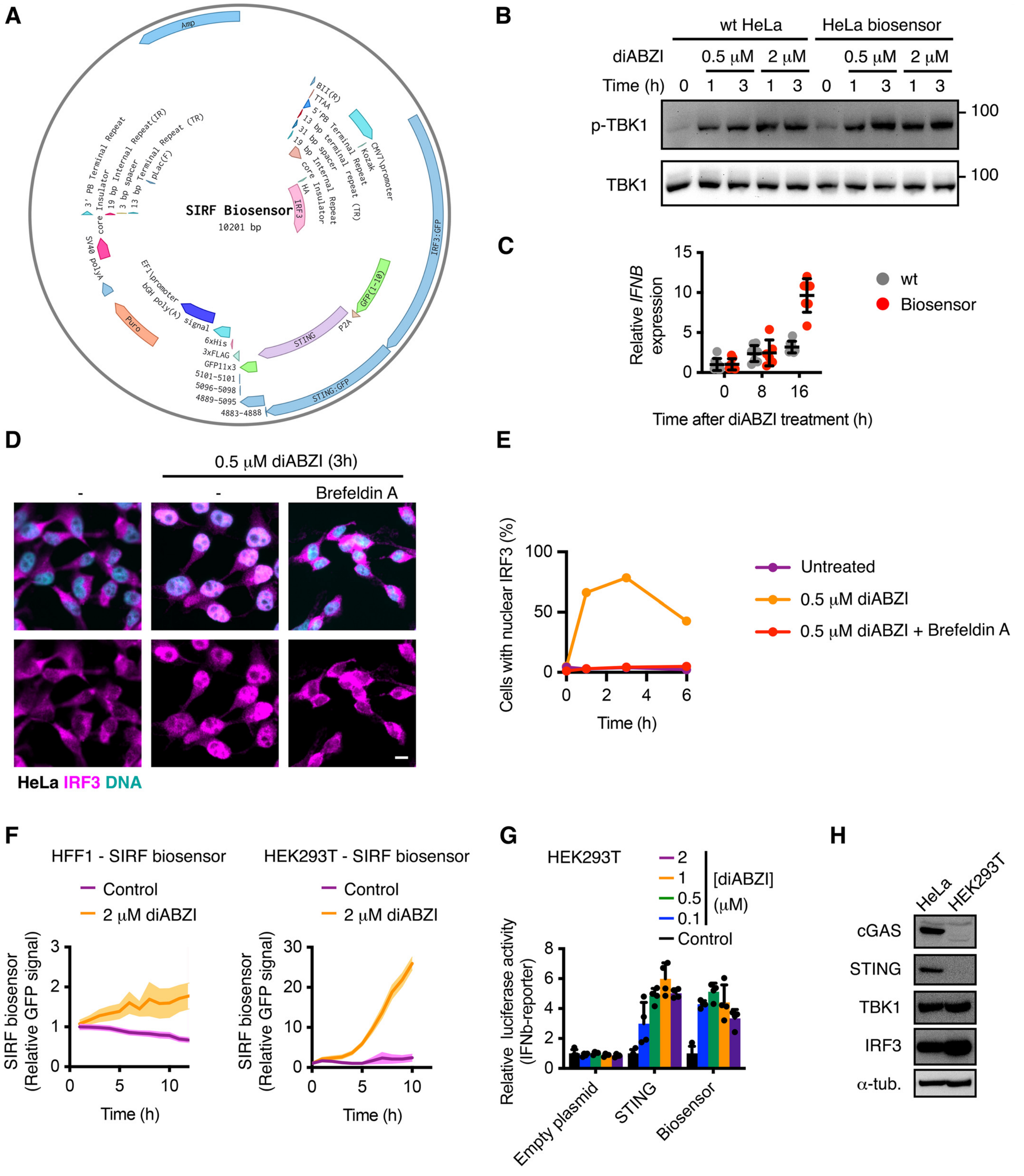
A
SIRF Biosensor
10201 bp
Amp
IRF3:GFP
STING:GFP
STING
P2A
GFP(1-10)
Puro
SV40 polyA
EF1\promoter
bGH poly(A) signal
6xHis
3xFLAG
GFP11x3
CMV7\promoter
Kozak
HA
IRF3
B
wt HeLa
HeLa biosensor
diABZI
0.5 μM
2 μM
Time (h)
0 1 3 1 3 0 1 3 1 3
p-TBK1
TBK1
100
C
Relative IFNB expression
Time after diABZI treatment (h)
wt
Biosensor
D
0.5 μM diABZI (3h)
-
Brefeldin A
HeLa IRF3 DNA
E
Cells with nuclear IRF3 (%)
Time (h)
Untreated
0.5 μM diABZI
0.5 μM diABZI + Brefeldin A
F
HFF1 - SIRF biosensor
HEK293T - SIRF biosensor
Control
2 μM diABZI
SIRF biosensor (Relative GFP signal)
Time (h)
G
HEK293T
[diABZI] (μM)
2
1
0.5
0.1
Control
Relative luciferase activity (IFNb-reporter)
Empty plasmid
STING
Biosensor
H
HeLa
HEK293T
cGAS
STING
TBK1
IRF3
α-tub.

 © The Author(s)

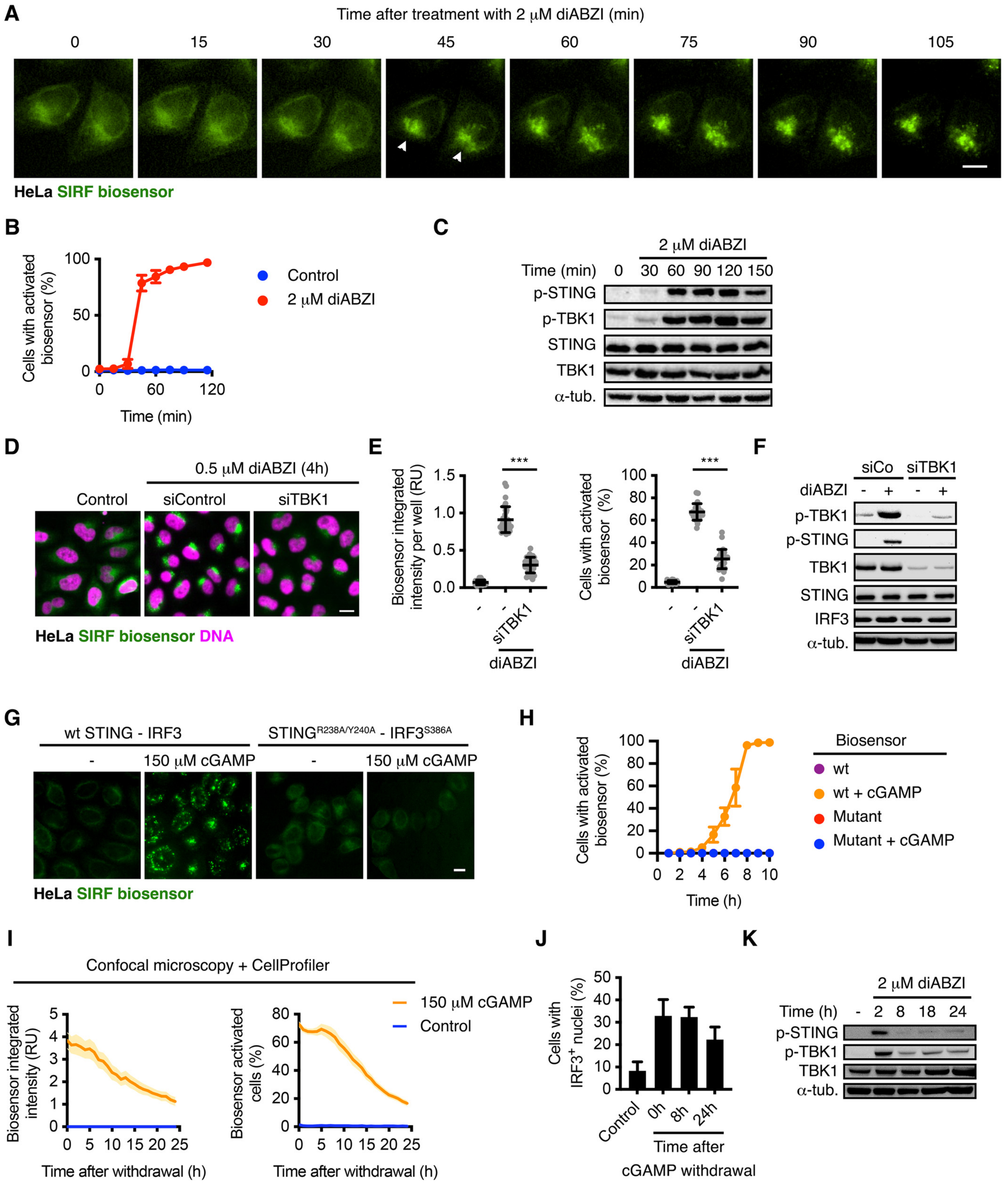
A
Time after treatment with 2 μM diABZI (min)
0 15 30 45 60 75 90 105
HeLa SIRF biosensor
B
Cells with activated biosensor (%)
100 50 0
0 60 120
Time (min)
Control
2 μM diABZI
C
2 μM diABZI
Time (min) 0 30 60 90 120 150
p-STING
p-TBK1
STING
TBK1
α-tub.
D
0.5 μM diABZI (4h)
Control siControl siTBK1
HeLa SIRF biosensor DNA
E
Biosensor integrated intensity per well (RU)
1.5 1.0 0.5 0.0
***
- - siTBK1
diABZI
Cells with activated biosensor (%)
100 80 60 40 20 0
F
siCo siTBK1
diABZI - + - +
p-TBK1
p-STING
TBK1
STING
IRF3
α-tub.
G
wt STING - IRF3
STING R238A/Y240A - IRF3 S386A
- 150 μM cGAMP - 150 μM cGAMP
HeLa SIRF biosensor
H
Cells with activated biosensor (%)
100 80 60 40 20 0
0 2 4 6 8 10
Time (h)
Biosensor
wt
wt + cGAMP
Mutant
Mutant + cGAMP
I
Confocal microscopy + CellProfiler
Biosensor integrated intensity (RU)
5 4 3 2 1 0
0 5 10 15 20 25
Time after withdrawal (h)
Biosensor activated cells (%)
80 60 40 20 0
150 μM cGAMP
Control
J
Cells with IRF3+ nuclei (%)
50 40 30 20 10 0
Control 0h 8h 24h
Time after cGAMP withdrawal
K
2 μM diABZI
Time (h) - 2 8 18 24
p-STING
p-TBK1
TBK1
α-tub.

© The Author(s)

Figure EV2. SIRF biosensor activation dynamics and requirements.

(**A**, **B**) Live cell confocal imaging analyses of HeLa SIRF biosensor cells in the presence or absence of 2 µM diABZI. In (**A**), arrows indicate the initial clustering of the biosensor. In (**B**), activation of the biosensor was scored as clustering of the GFP signal in 1 or more puncta, and it is represented as mean ± SEM of $N = 3$ independent time-lapses with >200 cells per condition. (**C**) Western blots of wt HeLa cells treated as in (**A**, **B**). (**D**, **E**) Immunofluorescence analyses of HeLa SIRF biosensor cells transfected with the indicated siRNAs and treated as indicated for 4 h. Note that cells were imaged in a 384x high content microscope prepared for screening and analysed using CellProfiler. Data represents mean ± SD of $N = 32$ different samples per condition, each analysed for % of active cells or integrated intensity (RU) using CellProfiler. In (**E**), P-values from one-way ANOVA between the indicated groups with $N = 32$ biological replicates are indicated as $^{***}P < 0.0001$ in both cases. (**F**) Western blots of HeLa cells treated as in (**D**, **E**). siCo, unrelated control siRNA siLRP6. (**G**, **H**) Live cell confocal imaging analyses of SIRF biosensor (wt) and STING(R238A/Y240A)-P2A-IRF3(S386A) (mutant) HeLa cells in the presence or absence of 150 µM cGAMP. In (**G**) activation of the biosensor was scored as clustering of the GFP signal in 1 or more puncta, and it is represented as mean ± SEM of $N = 3$ independent time-lapses with >200 cells per condition. (**I**) CellProfiler analyses of HeLa SIRF biosensor H2B-mCherry cells after live cell confocal imaging following cGAMP withdrawal (20-hour pre-treatment) or untreated. Data represent mean ± SEM of 8 different parallel time-lapses per condition each automatically analysed and with >200 cells. (**J**) Immunofluorescence analyses of wt HeLa cells after cGAMP withdrawal (20-hour pre-treatment) represented as mean ± SD of $N = 3$ biological replicates with >100 cells per condition (**K**) Western blots of wt HeLa cells treated as indicated. Scale bars = 10 µm. Source data are available online for this figure.

 © The Author(s)

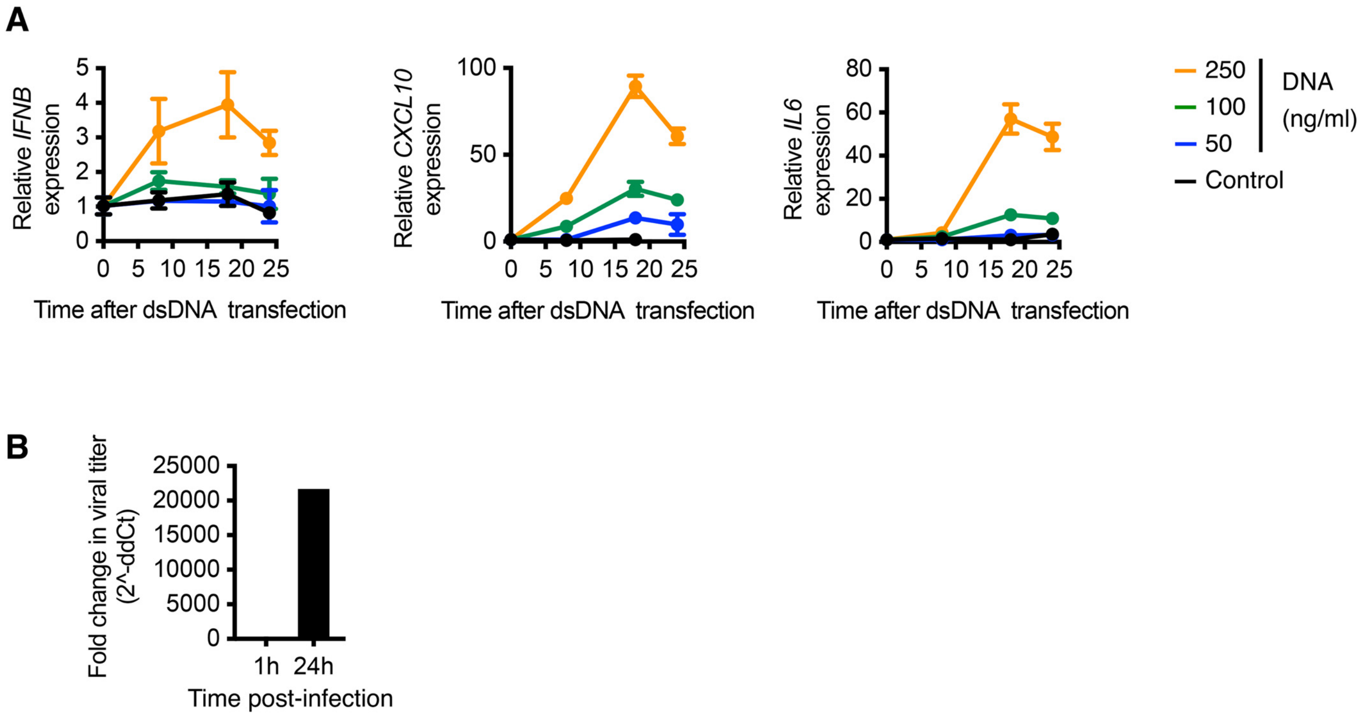

Figure EV3. Innate immune response to foreign dsDNA and dsRNA in HeLa cells.

(**A**) qRT-PCR analyses of wt HeLa cells treated as indicated and represented as mean ± SD of $N = 3$ biological replicates. (**B**) qRT-PCR analyses of influenza A viral RNA in infected HeLa SIRF biosensor cells, as indicated, and represented as mean of a representative experiment from $N = 3$ independent experiments. Source data are available online for this figure.

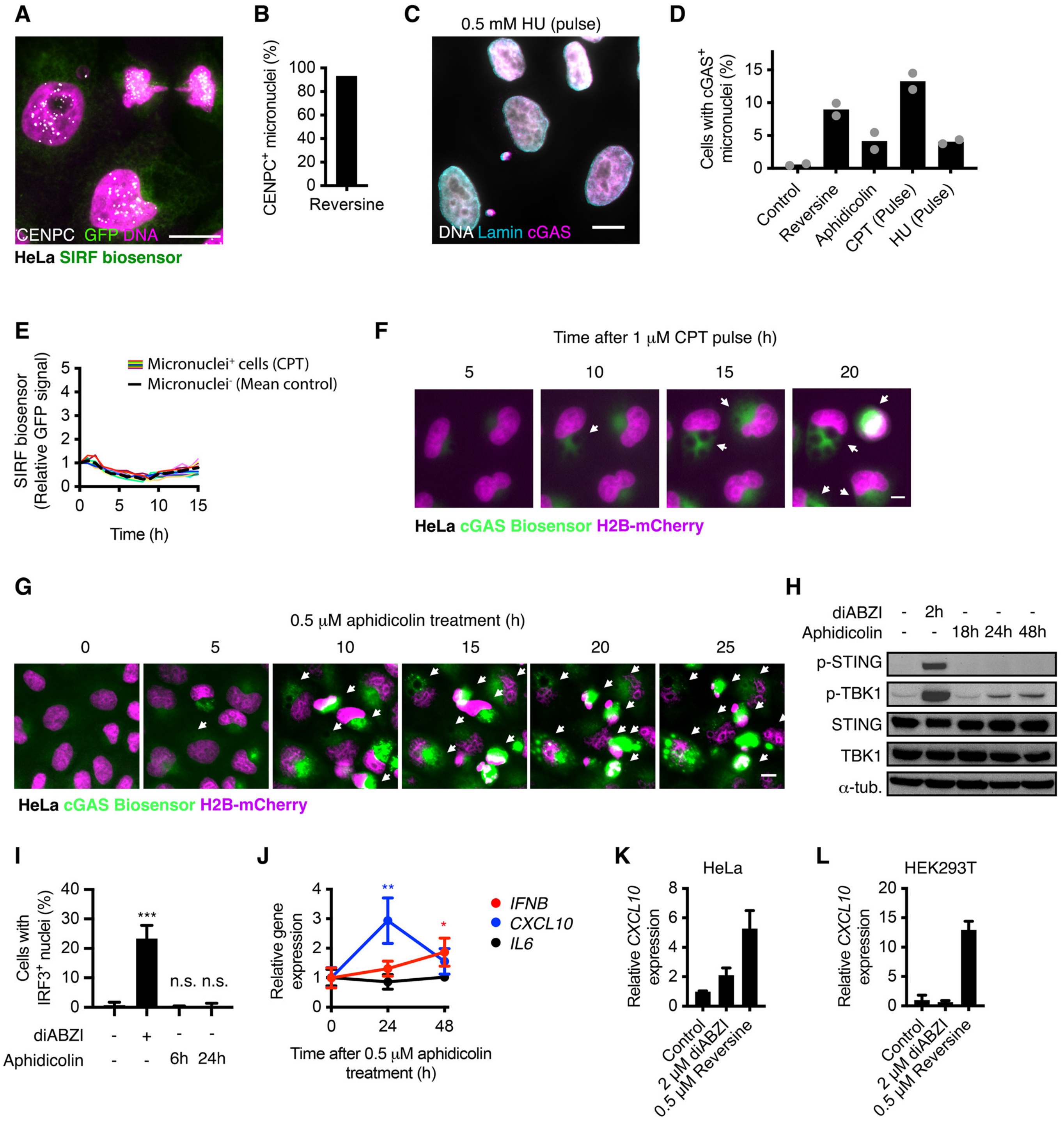
A
CENPC GFP DNA
HeLa SIRF biosensor
B
CENPC+ micronuclei (%)
Reversine
C
0.5 mM HU (pulse)
DNA Lamin cGAS
D
Cells with cGAS+ micronuclei (%)
Control
Reversine
Aphidicolin
CPT (Pulse)
HU (Pulse)
E
SIRF biosensor (Relative GFP signal)
Micronuclei+ cells (CPT)
Micronuclei- (Mean control)
Time (h)
F
Time after 1 μM CPT pulse (h)
5 10 15 20
HeLa cGAS Biosensor H2B-mCherry
G
0.5 μM aphidicolin treatment (h)
0 5 10 15 20 25
HeLa cGAS Biosensor H2B-mCherry
H
diABZI - 2h - - -
Aphidicolin - - 18h 24h 48h
p-STING
p-TBK1
STING
TBK1
α-tub.
I
Cells with IRF3+ nuclei (%)
***
n.s. n.s.
diABZI - + - -
Aphidicolin - - 6h 24h
J
Relative gene expression
**
*
IFNB
CXCL10
IL6
Time after 0.5 μM aphidicolin treatment (h)
K
HeLa
Relative CXCL10 expression
Control
2 μM diABZI
0.5 μM Reversine
L
HEK293T
Relative CXCL10 expression
Control
2 μM diABZI
0.5 μM Reversine

 © The Author(s)

Figure EV4. Characterisation of Micronuclei and replicative stress in HeLa cells.

(**A**, **B**) Immunofluorescence experiments in HeLa SIRF biosensor cells treated with 0.5 µM reversine for 18 h and stained as indicated. (**C**, **D**) Immunofluorescence analyses of HeLa cells treated with 2 µM diABZI for 4 h, with 0.5 µM reversine or 50 nM aphidicolin for 18 h, or pulsed for 4 h with 0.5 µM CPT or 0.5 mM HU followed by 18 h release. CPT, Topoisomerase inhibitor Camptothecin; HU, Hydroxyurea. In (**D**), data is mean of $N = 2$ independent experiments each with >200 cells per condition. (**E**, **F**) Live cell imaging analyses of HeLa SIRF biosensor H2B-mCherry cells pre-treated with a pulse of 0.5 µM CPT for 4 h and released for 18 h. In (**E**), single cells with micronuclei at the beginning of the recording were monitored for 15 h and are compared to the mean of control cells (dashed line). In (**F**), cells showing clear signs of autophagy and cell death are indicated with arrows. (**G**) Live cell imaging analyses of HeLa SIRF biosensor H2B-mCherry cells treated with 0.5 µM aphidicolin (DNA polymerase inhibitor). Cells showing clear signs of autophagy and cell death are indicated with arrows. (**H**) Western blots of wt HeLa cells treated as indicated. (**I**) Immunofluorescence analyses of wt HeLa cells treated as indicated. Data represents mean ± SD of $N = 4$ biological replicates with *P*-values from one-way ANOVA between from the indicated groups are indicated as ***$P < 0.0001$, or not significant (n.s.) from left to right as $P = 0.983$, $P = 0.999$. (**J**, **K**) qRT-PCR analyses of wt HeLa cells treated as indicated. Data represents mean ± SD of $N = 3$ biological replicates. *P*-values from one-way ANOVA compared to control for each gene are indicated as **$P = 0.0087$ for *CXCL10* and *$P = 0.049$ for *IFNB*. In (**K**), cells were treated with diABZI for 4 h or with reversine for 24 h. (**L**) qRT-PCR analyses of wt HEK29T cells treated with diABZI for 4 h or with reversine for 48 h. Data represents mean ± SD of $N = 3$ biological replicates. Scale bars = 10 µm. Source data are available online for this figure.

© The Author(s)

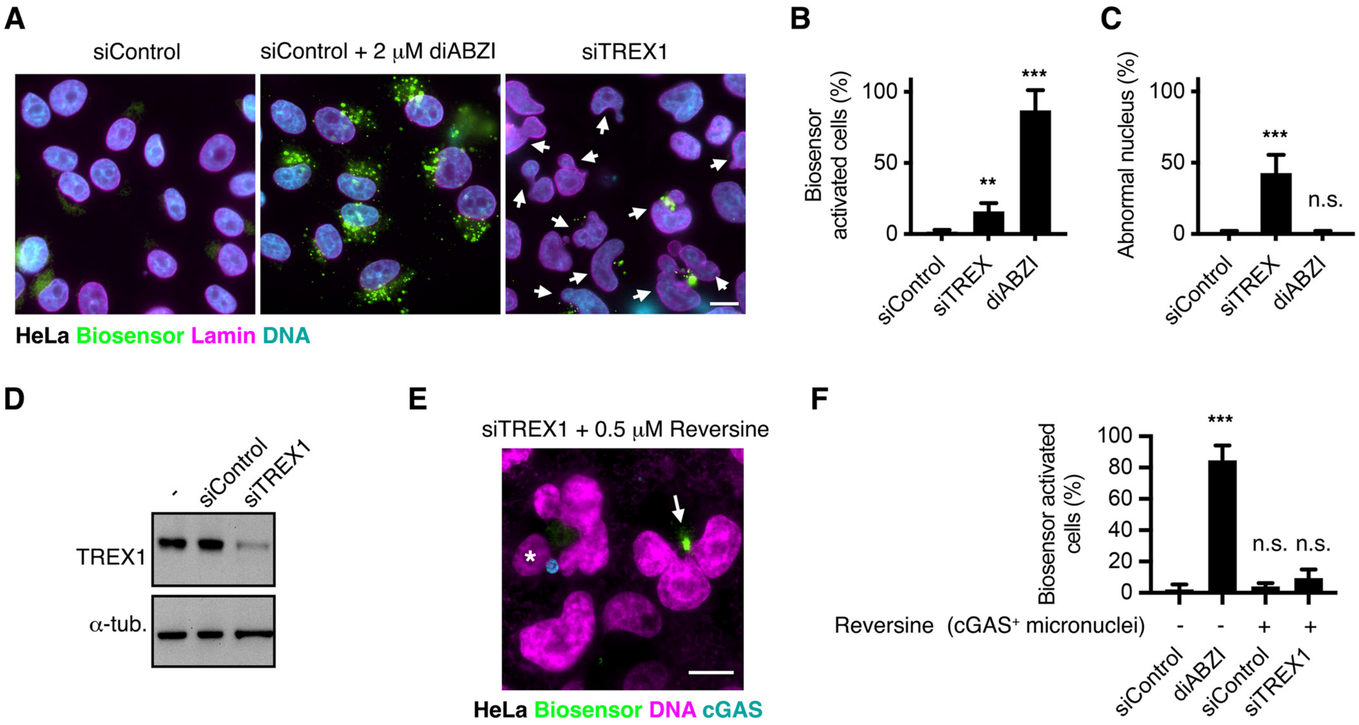

Figure EV5. SIRF biosensor activation upon TREX1 knock down.

(**A–C**) Immunofluorescence analyses of HeLa SIRF biosensor cells transfected with the indicated siRNAs for 72 h, or treated with 2 µM diABZI for 4 h. In (**C**), abnormal nuclear shape was scored in cells showing similar morphology as those pointed in (**A**). Data represents mean ± SD of $N = 8$–10 biological replicates with at least 25 cells per sample. *P*-values from one-way ANOVA between the indicated groups are indicated in (**B**) as $^{**}P = 0.0035$, and $^{***}P < 0.0001$, and in (**C**) as $^{***}P < 0.0001$ and n.s. = 0.9996. (**D**) Western blots of HeLa cells transfected as in (**A–F**). (**E, F**) Immunofluorescence analyses of HeLa SIRF biosensor cells transfected with the indicated siRNAs for 72 h, or treated with either 2 µM diABZI for 4 h or 0.5 µM reversin for 18 h. Note that reversine treated siTREX1 cGAS+ micronuclei cells (**F**) show less percentage of activation compared to cells only transfected with siTREX1 (**B**) suggesting that active biosensor in siTREX1 concentrate in cells with nuclear shape abnormalities and/or division failure. In (**F**), Data represents mean ± SD of $N = 3$ independent experiments. *P*-values from one-way ANOVA compared to control are indicated as $^{***}P < 0.0001$ and n.s. = 0.94 and 0.35, respectively. In (**A, E**) arrows indicate active biosensor upon siTREX1, while an asterisk marks a cGAS+ micronucleus. Scale bars = 10 µm. Source data are available online for this figure.

 © The Author(s)
